# S100A9 Derived from Chemoembolization‐Induced Hypoxia Governs Mitochondrial Function in Hepatocellular Carcinoma Progression

**DOI:** 10.1002/advs.202202206

**Published:** 2022-08-30

**Authors:** Chengrui Zhong, Yi Niu, Wenwu Liu, Yichuan Yuan, Kai Li, Yunxing Shi, Zhiyu Qiu, Keren Li, Zhu Lin, Zhenkun Huang, Dinglan Zuo, Zhiwen Yang, Yadi Liao, Yuanping Zhang, Chenwei Wang, Jiliang Qiu, Wei He, Yunfei Yuan, Binkui Li

**Affiliations:** ^1^ State Key Laboratory of Oncology in South China and Collaborative Innovation Center for Cancer Medicine Sun Yat‐Sen University Cancer Center Guangzhou 510030 China; ^2^ Department of Liver Surgery Sun Yat‐Sen University Cancer Center Guangzhou 510030 China; ^3^ Department of Gastric Surgery Sun Yat‐Sen University Cancer Center Guangzhou 510030 China; ^4^ Department of Anesthesiology Sun Yat‐sen University Cancer Center Guangzhou 510030 China

**Keywords:** chemoembolization, hepatocellular carcinoma, mitochondria, S100 calcium binding protein A9, transarterial chemoembolization

## Abstract

Transarterial chemoembolization (TACE) is the major treatment for advanced hepatocellular carcinoma (HCC), but it may cause hypoxic environment, leading to rapid progression after treatment. Here, using high‐throughput sequencing on different models, S100 calcium binding protein A9 (S100A9) is identified as a key oncogene involved in post‐TACE progression. Depletion or pharmacologic inhibition of S100A9 significantly dampens the growth and metastatic ability of HCC. Mechanistically, TACE induces S100A9 via hypoxia‐inducible factor 1α (HIF1A)‐mediated pathway. S100A9 acts as a scaffold recruiting ubiquitin specific peptidase 10 and phosphoglycerate mutase family member 5 (PGAM5) to form a tripolymer, causing the deubiquitination and stabilization of PGAM5, leading to mitochondrial fission and reactive oxygen species production, thereby promoting the growth and metastasis of HCC. Higher S100A9 level in HCC tissue or in serum predicts a worse outcome for HCC patients. Collectively, this study identifies S100A9 as a key driver for post‐TACE HCC progression. Targeting S100A9 may be a promising therapeutic strategy for HCC patients.

## Graphical abstract

S100 calcium binding protein‐A9 (S100A9) is associated with post‐transarterial chemoembolization (TACE) hepatocellular carcinoma (HCC) progression. TACE increases the expression of S100A9 in HCC cells. S100A9 promotes binding between ubiquitin specific peptidase‐10 (USP10) and phosphoglycerate mutase family member‐5 (PGAM5), leading to the stabilization of PGAM5, which increases reactive oxygen species levels and subsequently inducing the growth and metastasis of HCC.

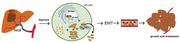

## 1 Introduction

Hepatocellular carcinoma (HCC) is the fourth leading cause of cancer‐related deaths worldwide, with a high recurrence and metastasis rate.^[^ ^1^, ^2^ ^]^ For many patients, there are no opportunity for surgery when they were diagnosed with HCC. According to American Association for the Study of Liver Diseases and European Association for the Study of the Liver HCC guidelines, Transarterial chemoembolization (TACE) is the recommended therapy for patients with advanced HCC.^[^ ^3^, ^4^ ^]^ TACE involves intra‐arterial infusion of chemotherapeutic drugs such as doxorubicin followed by selective blocking of the arterial blood supply to the cancer, resulting in ischemia and hypoxic necrosis of HCC cells. However, the blood supply blockade may also induce a hypoxic condition that promotes tumor progression, metastasis, and therapeutic drug resistance via a myriad of cell activities in both tumor and stromal cells. Hypoxia stabilizes HIF1A, which is involved in the growth and metastasis of HCC by activating the transcription of genes, such as vascular endothelial growth factor, that are crucial to cancer metastasis and recurrence.^[^ ^5^, ^6^ ^]^ Previous studies have shown that TACE can promote local recurrence and lung metastasis of HCC.^[^ ^7^, ^8^ ^]^ To develop an effective approach for predicting the survival benefits from TACE, it is essential to understand the mechanisms of post‐TACE progression.

S100 calcium binding protein A9 (S100A9) is a member of the S100 protein family. It is a calcium‐binding protein that was first identified as a proinflammatory mediator released by bone marrow‐derived cells in response to cell damage, infection, or inflammation.^[^ ^9^, ^10^ ^]^ Recent studies have shown that S100A9 is also involved in carcinogenesis and tumor progression and inhibits the tumor immune response.^[^ ^11^, ^12^, ^13^ ^]^ S100A9 is upregulated and correlated with poor differentiation in HCC.^[^ ^14^ ^]^ S100A9 might activate the reactive oxygen species (ROS)‐dependent signaling pathways and protect against cell death.^[^ ^15^ ^]^ However, its intrinsic mechanisms remain underexplored. Therefore, the role of S100A9 in the initiation and progression of HCC and the mechanisms regarding its role post‐TACE remain largely unclear.

Mitochondria, the most important source of ROS, play important and diverse roles in regulating cell behavior, from regulating proliferation and survival to promoting oxidative damage and cell death. In most tumor cells, ROS are elevated and act as essential signaling molecules in the regulation of cancer initiation and development.^[^ ^16^, ^17^ ^]^ Increasing evidence shows that mitochondrial dynamics play important roles in different kinds of cells.^[^ ^18^, ^19^ ^]^ However, how mitochondrial dynamics are integrated into ROS‐related signaling pathways involved in HCC cell survival remains largely unknown.

In the present study, we investigated the role of S100A9 in post‐TACE HCC progression and its underlying mechanisms. We provided the first evidence that hypoxia induced by TACE could increase the expression of S100A9 in HCC. Targeting S100A9 with Tasquinimod (Tas) significantly inhibited the growth and metastasis of HCC in vivo and in vitro. S100A9 promotes binding between ubiquitin specific peptidase 10 (USP10) and phosphoglycerate mutase family member 5 (PGAM5), leading to the deubiquitination and stabilization of PGAM5, which increases mitochondrial ROS levels by promoting mitochondrial fission and subsequently inducing the growth and metastasis of HCC. Additionally, elevated serum S100A9 levels in patients who received preoperative TACE were associated with HCC progression. Our study reveals that S100A9 is a key driver of post‐TACE HCC progression. Targeting S100A9 could be a promising therapeutic strategy for treating HCC.

## 2 Results

### 2.1 Transcriptome Sequencing and CRISPR/Cas9 Screening Identify S100A9 as a Key Driver of Post‐TACE HCC Progression

**Figure 1:**
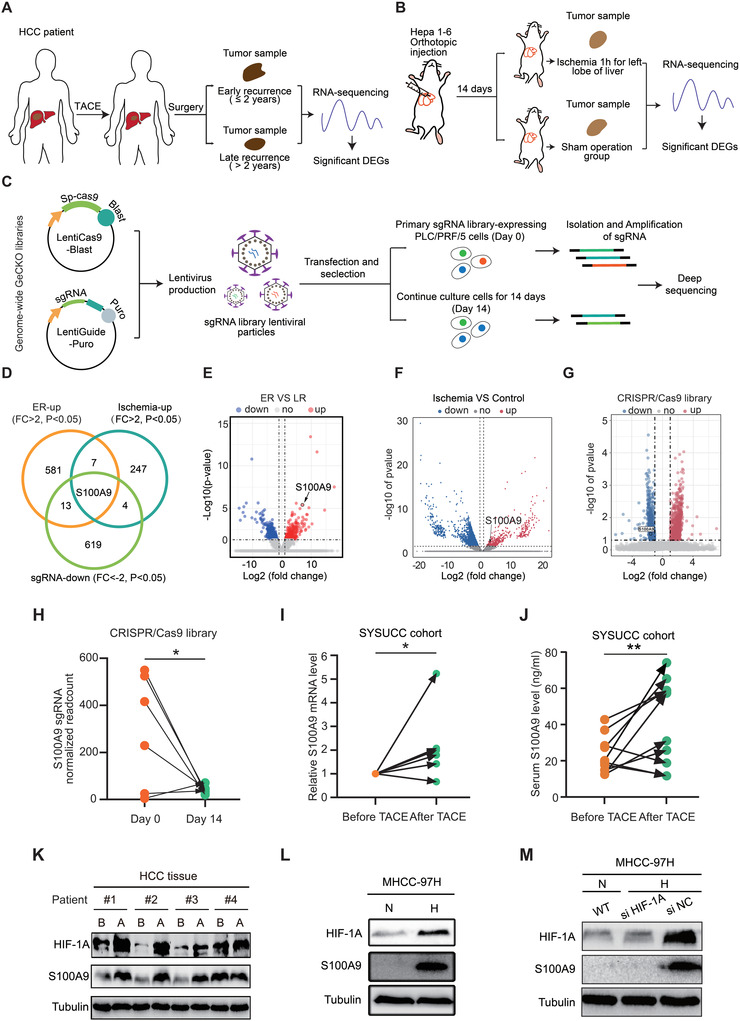
S100A9 is a key driver of post‐TACE HCC progression. A) Schematic of the screening pattern of TACE‐related ER genes by RNA sequencing. B) Schematic of the experimental strategy used to identify hypoxia‐related genes in vivo (nude mice). C) Schematic diagram of the genome‐wide CRISPR/Cas9 knockout library screen for HCC cells. D) Venn diagram summarizing the results relative to the identification of a set of genes associated with TACE, ischemia, and CRISPR/Cas9 library screening. E) Volcano plot showing that S100A9 is one of the most upregulated genes in the HCC tissues of patients with early recurrence. F) Volcano plot showing the DEGs between the ischemia and control groups. S100A9 was one of the upregulated genes in the ischemia group. G) Volcano plot showing the DEGs between day 0 and day 14 associated with CRISPR/Cas9 library screening in PLC/PRF/5 cells. S100A9 was one of the downregulated genes on day 14. H) Quantification of S100A9 sgRNA readcount levels on day 0 and day 14 associated with the CRISPR/Cas9 library screen (*n* = 6). I) RNA levels of S100A9 in the HCC tissues before and after TACE in the same patient (*n* = 6). J) Serum S100A9 levels before and after TACE In the same patient (*n* = 10). Data in (H)–(J) are presented as mean ± SEM, *p < 0.05, **p < 0.01, by two‐tailed paired Student *t*‐test. K) Protein levels of HIF1A and S100A9 in the HCC tissues before and after TACE in the same patient. (B) before TACE, (A) after TACE. L) Hypoxia (1% oxygen 24 h) stabilized HIF1A and induced S100A9 expression in MHCC‐97H cells. (N) normal oxygen, (H) hypoxia. M) Silencing HIF1A suppressed the expression of S100A9 induced by hypoxia in MHCC‐97H cells.

To explore the key gene that affects post‐TACE HCC progression, three sets of independent screening were performed. First, we collected early‐recurrence (ER) and late‐recurrence (LR) HCC tumor sample tissues from patients who received preoperative TACE. The significantly upregulated genes in the ER group were defined as associated with TACE ER (Fold change [FC] > 2, *p* < 0.05). Next, to simulate a clinical hypoxic tumor environment after TACE treatment, mouse liver tumor samples using an ischemia model were collected for transcriptome sequencing analysis. The significantly upregulated genes in the ischemia group were the genes that induce hypoxia (FC > 2, *p* < 0.05). Then, genome‐wide CRISPR/Cas9 knockout library screening was performed. Cells carrying this library were cultured for 14 days to screen for genes that affected HCC growth. Single‐guide RNA (sgRNA) targeting growth‐related genes were negatively selected in the knockout mutant cell pool (FC < −2, *p* < 0.05), (**Figure** **1**A–C). Our results showed that 602 differently expressed genes (DEGs) were upregulated in the ER group compared with the LR group. Cancer‐related pathways such as cell adhesion molecules and the PI3K‐AKT signaling pathway were enriched in the ER group (Figure 1D,E and Figure S1A,B, Supporting Information), and 259 upregulated DEGs were identified in the ischemia group compared with the control group (Figure 1F and Figure S1C, Supporting Information). A subset of sgRNAs targeting 637 genes was significantly depleted in the CRISPR/Cas9 library screening (Figure 1G and Figure S1D, Supporting Information).

After overlapping three sets of screening data, we identified that S100A9 is a hypoxia‐induced HCC oncogene that is closely associated with early recurrence after TACE treatment. (Figure 1D–G).

### 2.2 TACE Induces Hypoxia‐inducible Factor 1α(HIF1A)‐Mediated S100A9 Expression in HCC

To verify whether TACE induces S100A9 expression, we compared the S100A9 levels in HCC tissues and serum before and after preoperative TACE in each patient. TACE was associated with an increase in S100A9 expression in both the HCC tissues and in the serum of most patients (Figure 1H–J). HCC tissues contain both tumor cells and immune cells, with immune cells reported to express S100A9. Flow cytometry results showed that ≈65% of the S100A9‐positive cells were EPCAM‐positive cells. The results also showed an increase in the proportion of S100A9 expression in EPCAM‐positive cells and a decrease in CD45‐positive cells after TACE treatment (Figure S1E,F, Supporting Information). Multiple immunofluorescence (MIF) staining showed the same result (Figure S1G–J, Supporting Information).

TACE treatment includes embolization and chemotherapy. We therefore wondered which treatment caused the upregulation of S100A9. Both in vivo and in vitro experiments showed that ischemia and hypoxia (1% oxygen, 24 h) may be the cause of elevated S100A9 (Figure S1K,L, Supporting Information). Western blot analysis showed that TACE, and hypoxia both increased HIF1A and S100A9 expression in HCC tissues and cell lines, respectively (Figure 1K,L and Figure S1M, Supporting Information). Silencing HIF1A suppressed the expression of S100A9 induced by hypoxia (Figure 1M and Figure S1N, Supporting Information), indicating that TACE increases S100A9 expression through HIF1A. Luciferase reporter assays revealed a marked decrease in luciferase activity in the P (+350/+900) construct compared with the other three constructs (Figure S1O,P, Supporting Information). Chromatin immunoprecipitation and semiquantitative PCR analyses confirmed that HIF1A physically binds to the S100A9 promoter (Figure S1Q,R, Supporting Information). Collectively, these results confirm that S100A9 is derived from TACE‐induced hypoxia in HCC.

### 2.3 S100A9 Promotes the Growth and Metastasis of HCC Cells In Vitro and In Vivo

**Figure 2:**
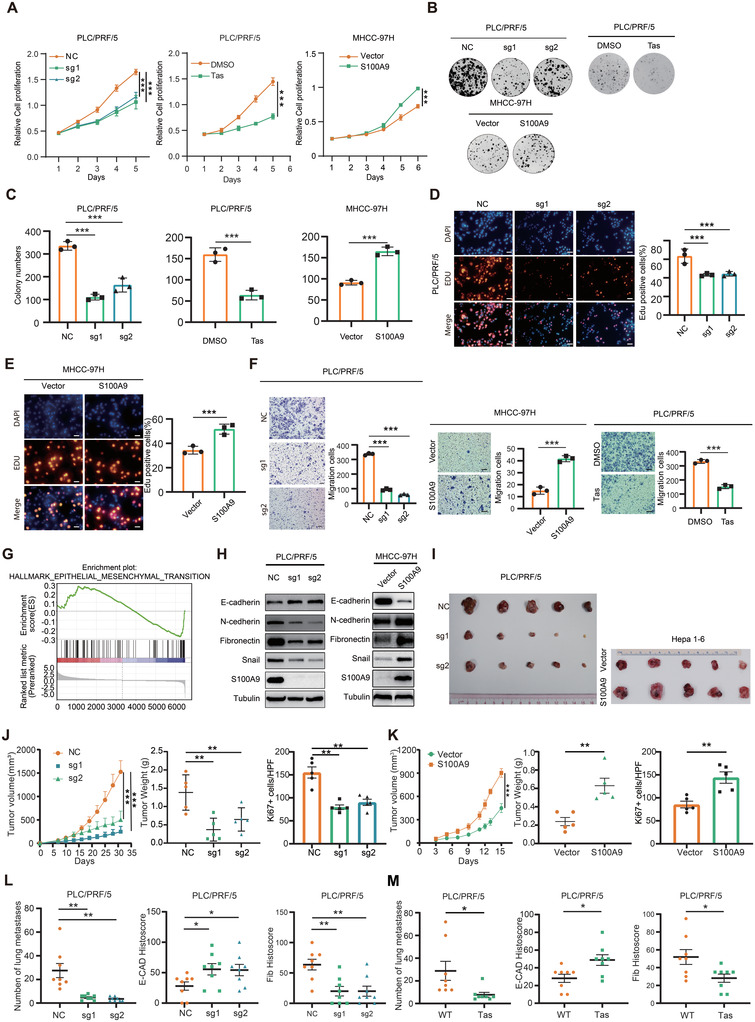
S100A9 promotes the growth and metastasis of HCC cells in vitro and in vivo. A–E) S100A9 knockout or Tas administration decreased and S100A9 overexpression promoted the proliferation of HCC cells, as indicated by CCK‐8 assay (A), clone formation assay (B,C) and EdU assay (D,E). Scale bars in (D) and (E) are 100 µm. F) S100A9 knockout or administration of 100 µm of Tas decreased and S100A9 overexpression promoted the migration of HCC cells. Scale bars = 100 µm. Data in (A)–(F) are presented as mean ± SEM, *n* = 3. ***p < 0.001 by two‐tailed unpaired Student *t*‐test. G) Gene set enrichment analysis indicated a significant change in EMT signaling induced by S100A9. H) Western blot analysis of S100A9 and EMT markers in HCC cells. I–K) Knockout of S100A9 dramatically suppressed tumor growth, while overexpression of S100A9 promoted tumor growth in vivo, as indicated by the tumor growth rate, tumor weight, and quantification of Ki67+ stained cells in tumors (*n* = 5). L,M) Knockout of S100A9 or Tas administration suppressed the lung metastatic capacity of HCC cells and revealed an increase in E‐cadherin expression and a reduction in fibronectin expression in lung metastases (*n* = 8). Data in (J)–(M) are presented as mean ± SEM, *p < 0.05, **p < 0.01, ***p < 0.001 by two‐tailed unpaired Student *t*‐test.

To investigate the functional roles of S100A9 in HCC cells, stable S100A9‐knockout/overexpression HCC cells were constructed (Figure S2A–C, Supporting Information). Tas, a drug with selective neutralizing activity against S100A9 (Figure S2D, Supporting Information), was used to identify the function of S100A9. Knockout of S100A9, and Tas treatment inhibited the proliferation and migration of HCC cells, and overexpression of S100A9 promoted the proliferation and migration of HCC cells. (**Figure** **2**A–F and Figure S2E–J, Supporting Information). RNA sequencing and gene set enrichment analysis revealed that DEGs between S100A9 wild‐type and S100A9 knockdown cells were involved in the epithelial–mesenchymal transition (EMT) pathway, which was confirmed by Western blotting (Figure 2G,H and Figure S2K, Supporting Information). These results indicate that S100A9 promotes the proliferation and migration of HCC cells in vitro.

To further explore the oncogenic role of S100A9 in vivo, a subcutaneous tumor model was generated. Knockout of S100A9 significantly delayed tumor progression. In contrast, overexpression of S100A9 remarkably enhanced tumor growth (Figure 2I–K). Next, an experimental lung metastasis model was established following tail vein injection. Overexpression of S100A9 enhanced the metastatic ability of tumor cells, even after neutralizing CD11b‐positive cells in a C57 mouse model, as measured by in vivo bioluminescent imaging (Figure S2L,M, Supporting Information). In the nude mouse model, compared with S100A9 null cells, S100A9 wild‐type HCC cells showed a clear increase in lung colonization capacity, which was decreased following Tas treatment in mice. Consistent with the results in cell lines, decreased E‐cadherin and increased fibronectin expression were detected in S100A9‐null lung lesions. This response was reversed by Tas (Figure 2L,M and Figure S2N–T, Supporting Information). These results indicate that S100A9 promotes the growth and metastatic ability of HCC in vivo.

### 2.4 S100A9 Modulates Cellular Mitochondrial Function and Promotes ROS Production

**Figure 3:**
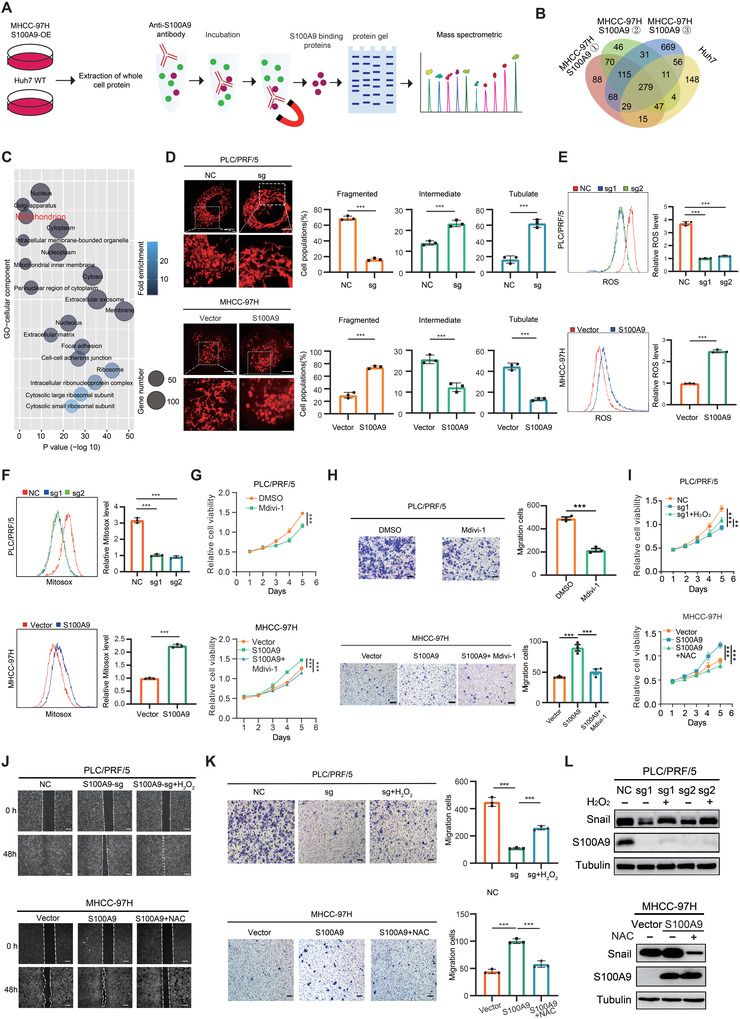
S100A9 modulates cellular mitochondrial function and promotes ROS production. A) Co‐IP procedure in HCC cells. B) Venn diagram showing the proteins binding with S100A9 identified by LC–MS. C) Gene ontology analysis of proteins binding with S100A9 identified by LC–MS. D) S100A9 knockout inhibited while S100A9 overexpression promoted mitochondrial fission in HCC cells. Scale bars = 5 µm. The proportion of HCC cells (*n* = 100 cells for each sample, repeated three times) with tubulated, intermediate, and fragmented mitochondria was quantified. E,F) S100A9 knockout reduced whereas S100A9 overexpression increased intracellular ROS (E) or intramitochondrial ROS (F) production in HCC cells, as indicated by DCFH‐DA or MitoSOX fluorescence assays. G,H) Mdivil‐1 (10 µm) inhibited the proliferation (G) and migration ability (H) in HCC cells. Scale bars = 100 µm. I) Enhanced ROS production with 100 µm H_2_O_2_ (top) promoted while reduced ROS production with 30 mm NAC (bottom) inhibited the proliferation of HCC cells, as indicated by the CCK‐8 assay. J,K) Enhanced ROS production with 100 µm H2O2 (top) promoted while reduced ROS production with 30 mm NAC (bottom) for 12 h inhibited the migration of HCC cells, as indicated by wound‐healing assay (J) and transwell assay (K). Scale bars = 100 µm. L) Western blot analysis for the expression of Snail in HCC cells treated as indicated. Data in (E)–(I) and (K) are presented as mean ± SEM, *n* = 3. **p < 0.01, ***p < 0.001 by two‐tailed unpaired Student *t*‐test.

To delineate the function of S100A9 in HCC, co‐immunoprecipitation (Co‐IP) and liquid chromatography–mass spectrometry (LC–MS) were performed to screen for the proteins interacting with S100A9 (**Figure** **3**A,B). Functional enrichment analysis showed that S100A9 was involved in the regulation of multiple pathways in HCC cells, among which, the mitochondrial functional pathway was closely related to hypoxia and cell energy metabolism (Figure 3C). Therefore, we first explored the effect of S100A9 on mitochondrial morphology. MitoTracker staining indicated that mitochondrial elements became significantly elongated in S100A9 null cells compared with the control group, while overexpression of S100A9 resulted in a remarkable fragmentation of mitochondria (Figure 3D). Mitochondrial fission which is usually associated with ROS production indicated that the knockout of S100A9 or Tas treatment decreased the ROS level of HCC cells according to flow cytometry (Figure 3E and Figure S3A–D, Supporting Information). Furthermore, the use of MitoSOX, a specific reagent for detecting the level of ROS in mitochondria, revealed that S100A9 promoted mitochondrial ROS production (Figure 3F). Mdivi‐1, a mitochondrial fission inhibitor, significantly inhibited ROS levels in HCC cells, while it could suppress the proliferation and invasive ability of HCC. This indicated that increased mitochondrial fission induced by S100A9 promoted ROS production (Figure 3G,H and Figure S3E,F, Supporting Information). However, S100A9 did not affect the oxidative phosphorylation of HCC cells, but enhanced glycolysis rate and ATP production of HCC cells (Figure S3G–I, Supporting Information). NADPH oxidase (NOX) is another important source of ROS production in cancer cells.^[^ ^20^ ^]^ However, no consistent change in NOX RNA levels in S100A9 knockout or overexpression cells was found (Figure S3J,K, Supporting Information).

To verify if S100A9 was associated with elevated ROS levels, HCC cells were treated with H_2_O_2_, N‐acetyl‐L‐cysteine (NAC, ROS scavenger),^[^ ^21^ ^]^ or Mito‐tempo (intramitochondrial ROS scavenger)^[^ ^22^, ^23^ ^]^ to change cellular or mitochondrial ROS levels. H_2_O_2_ treatment significantly promoted the growth and migration of HCC cells suppressed by S100A9 knockout. While NAC or Mito‐tempo treatment dampened the growth and migration of HCC cells induced by S100A9 (Figure 3I–L and Figure S3L–N, Supporting Information). This demonstrates that increased mitochondrial fission and subsequent ROS production were involved in the oncogenic effect of S100A9 in HCC.

### 2.5 S100A9 Acts as a Scaffold Protein to Recruit USP10 and PGAM5 to Maintain PGAM5 Stability

**Figure 4:**
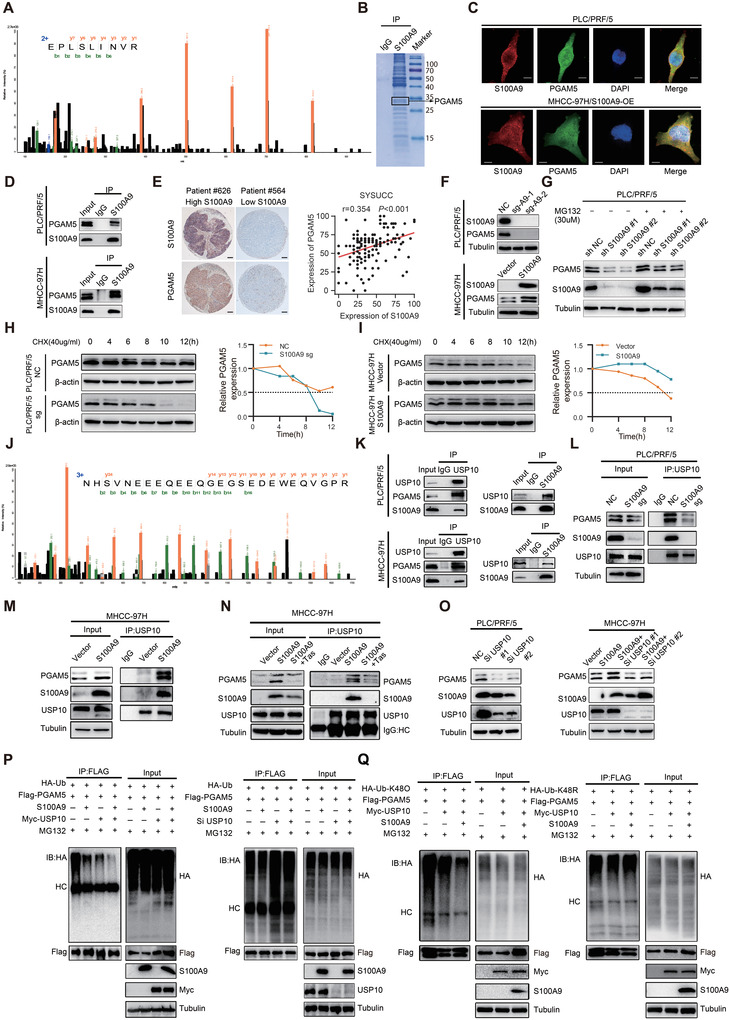
S100A9 recruits USP10 and PGAM5 to maintain PGAM5 stability. A) Fragmentation spectrum of the peptide of PGAM5 identified by LC–MS. B) Identified PGAM5 peptides are shown using Coomassie bright blue staining. C) Representative images of immunofluorescence colocalization of S100A9 (red) and PGAM5 (green) in PLC/PRF/5 (top) and MHCC‐97H (bottom) cells. Scale bars = 5 µm. D) Co‐IP showing the binding of S100A9 and PGAM5 in HCC cells. E) Representative IHC staining images of S100A9 and PGAM5 expression in HCC tissues (left) and positive correlations between S100A9 and PGAM5 protein levels in HCC tissues based on IHC scores (right). Scale bar = 100 µm (SYSUCC cohort, *n* = 172. *r* and *P* value were measured using Pearson's correlation test). F) Knockout of S100A9 decreased and overexpression of S100A9 increased the protein level of PGAM5 in HCC cells. G) Western blot analysis showing PGAM5 and S100A9 levels in PLC/PRF/5 cells that were treated with 30 µm MG132 for 6 h. H) Western blotting (left) and quantification of (right) PGAM5 protein levels in PLC/PRF/5/S100A9‐sg or control cells that were treated with 40 µg mL^−1^ CHX for the indicated times. I) Western blotting (left) and quantification of (right) PGAM5 protein levels in MHCC‐97H/S100A9‐overexpressing or control cells that were treated with 40 µg mL^−1^ CHX for the indicated times. J) Fragmentation spectrum of the USP10 peptide identified by LC–MS. K) S100A9, USP10, and PGAM5 formed a ternary complex in HCC cells. L) Knockout of S100A9 inhibited the binding of USP10 and PGAM5 in PLC/PRF/5 cells. M) Overexpression of S100A9 promoted the binding of USP10 and PGAM5 in MHCC‐97H cells. N) Tas treatment inhibited the binding of USP10 and PGAM5 in HCC cells. O) Knockdown of USP10 decreased PGAM5 protein levels in HCC cells. P) Coexpression of USP10 and S100A9 significantly decreased PGAM5 ubiquitination, while depletion of USP10 increased PGAM5 ubiquitination. Q) USP10 decreased Lys‐K48‐linked polyubiquitination of PGAM5 but had little effect on Lys‐K48R‐linked polyubiquitin chains.

To further explore the mitochondria‐related genes affected by S100A9, we analyzed the LC–MS results and found that S100A9 binds to PGAM5 (**Figure** **4**A). Previous studies have shown that PGAM5 was closely related to the mitochondrial fission process, thus causing an increase in ROS.^[^ ^19^, ^24^ ^]^ In the present study, the knockdown of PGAM5 significantly promoted dephosphorylation at Ser637 of dynamin‐related protein 1. This changed the mitochondrial morphology from fragmented to elongated and resulted in a reduction in intracellular ROS levels and intramitochondrial ROS levels, thereby inhibiting the growth and migration of HCC cells (Figure S4A–G, Supporting Information). These results indicate that PGAM5 promotes ROS via the mitochondrial pathway in HCC.

As indicated by Co‐IP and immunofluorescence, PGAM5 binds to S100A9 (Figure 4B–D and Figure S4H,I, Supporting Information). Immunohistochemistry (IHC) revealed a positive correlation between S100A9 and PGAM5 protein levels based on IHC scores (Figure 4E). Interestingly, knockout or overexpression of S100A9 was associated with a change in the protein level but not the mRNA level of PGAM5 (Figure 4F and Figure S4J,K, Supporting Information), indicating that S100A9 regulated PGAM5 levels by maintaining the stability of the PGAM5 protein. To validate whether S100A9 affected PGAM5 expression in a proteasome‐dependent manner, cells with or without S100A9 knockdown were treated with the proteasome inhibitor MG132. MG132 reversed the downregulation of PGAM5, even though S100A9 was depleted (Figure 4G). Knockout of S100A9 accelerated PGAM5 degradation in the presence of cycloheximide (CHX, a protein synthesis inhibitor) and the overexpression of S100A9 delayed PGAM5 degradation in the presence of CHX (Figure 4H,I).

S100A9 did not directly degrade proteins, raising the possibility that S100A9 is a scaffold protein recruiting functional proteins to bind with PGAM5, thus affecting PGAM5 stability. The LC–MS results showed that S100A9 binds to USP10 (Figure 4J). Co‐IP confirmed that S100A9 binds to both PGAM5 and USP10, while USP10 also binds to S100A9 and PGAM5 (Figure 4K), suggesting that S100A9, PGAM5, and USP10 may assemble into a ternary complex. The amount of the USP10/PGAM5 complex associated with high S100A9 expression levels was higher in HCC cells with low S100A9 expression levels, indicating that the binding between USP10 and PGAM5 mostly relies on S100A9 (Figure 4L–N). Silencing USP10 in HCC cells significantly decreased the protein level of PGAM5 (Figure 4O and Figure S4M, Supporting Information), suggesting that USP10 maintained the protein level of PGAM5 and exerted such regulation through the deubiquitination of PGAM5. As expected, co‐transfection of 293T cells with S100A9 and USP10 dramatically decreased PGAM5 ubiquitination, while depletion of USP10 increased PGAM5 ubiquitination (Figure 4P). This shows that USP10 is an essential deubiquitinating enzyme for PGAM5. We wonder whether the Lys‐48‐ linked polyubiquitin chain on PGAM5 is modulated by USP10. Co‐transfection of USP10 and S100A9 significantly decreased Lys‐K48‐linked polyubiquitination on PGAM5 but imposed little effect on Lys‐K48R‐linked polyubiquitin chains, suggesting that the Lys‐K48‐linked ubiquitin chains may be removed by USP10 (Figure 4Q).

### 2.6 S100A9 Governs Mitochondrial Function and HCC Progression through a PGAM5‐Dependent Pathway

**Figure 5:**
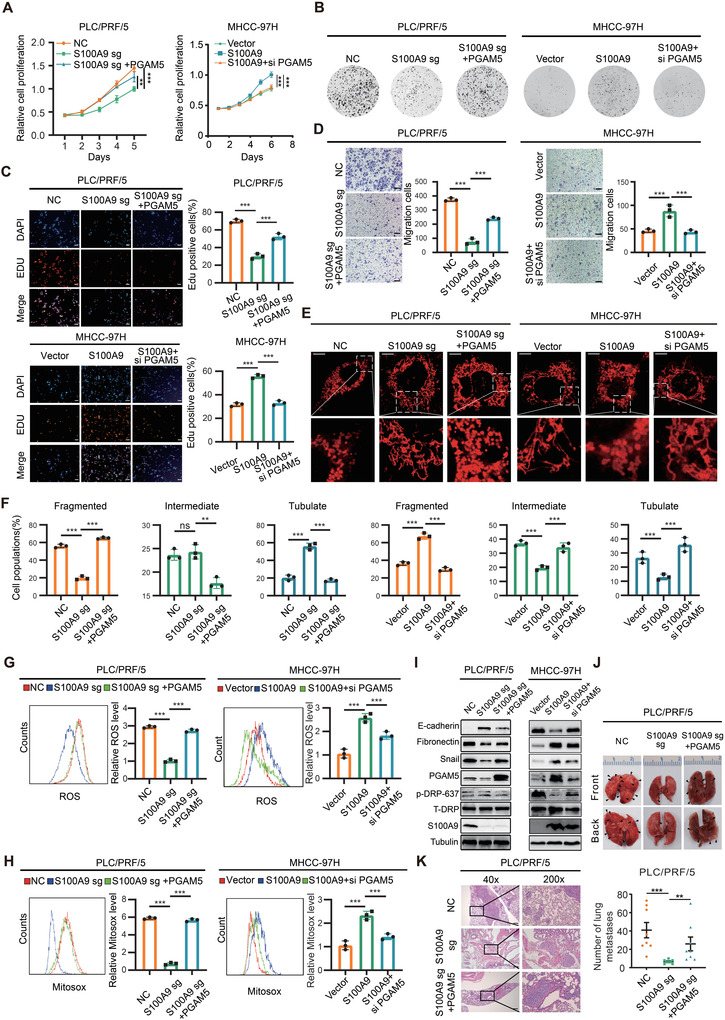
S100A9 affects HCC growth and metastasis through a PGAM5‐dependent pathway. A–C) Ectopic expression of PGAM5 enhanced S100A9 knockout‐induced cell growth arrest, while knockdown of PGAM5 attenuated S100A9‐induced cell growth, as indicated by CCK‐8 assay (A), clone formation assay (B), and EdU assay (C). Scale bars = 100 µm. D) Ectopic expression of PGAM5 enhanced S100A9 knockout‐induced cell migration, while knockdown of PGAM5 attenuated S100A9‐induced cell migration, as indicated by Transwell assay. Scale bars = 100 µm. Data in (A)–(D) are presented as mean ± SEM, *n* = 3. **p < 0.01, ***p < 0.001 by two‐tailed unpaired Student *t*‐test. E,F) Ectopic expression of PGAM5 reversed S100A9‐sg‐induced mitochondrial fusion and promoted mitochondrial fission, while knockdown of PGAM5 inhibited S100A9‐induced mitochondrial fission. Scale bars = 5 µm. The proportion of HCC cells (*n* = 100 cells for each sample, repeated three times) with tubulated, intermediate, and fragmented mitochondria was quantified. Data are presented as mean ± SEM, **p < 0.01, ***p < 0.001 by two‐tailed unpaired Student *t*‐test. G,H) Ectopic expression of PGAM5 enhanced the intracellular ROS level of S100A9‐sg cells, while knockdown of PGAM5 attenuated S100A9‐induced intracellular ROS (G) and intramitochondrial ROS (H), as indicated by DCFH‐DA and MitoSOX fluorescence assays (*n* = 3). I) Western blot analysis showing the expression of S100A9, EMT marker, and PGAM5 target genes in HCC cells. J,K) Ectopic expression of PGAM5 promoted the lung metastatic capacity of HCC cells (*n* = 8). Scale bars = 100 µm. Data in (G), (H), and (K) are presented as mean ± SEM, **p < 0.01, *** *p* < 0.001 by two‐tailed unpaired Student *t*‐test.

To assess whether S100A9 promotes tumorigenesis through a PGAM5‐dependent pathway, stable PGAM5‐overexpressing/knockdown HCC cells were constructed (Figure S5A–C, Supporting Information). Ectopic expression of PGAM5 reversed S100A9 knockout‐induced cell growth arrest, while silencing PGAM5 inhibited S100A9‐induced cell growth and migration (**Figure** **5**A–D and Figure S5D,E, Supporting Information). PGAM5 was also responsible for the mitochondrial fission and ROS production induced by S100A9 (Figure 5E–H and Figure S5F,G, Supporting Information). Likewise, PGAM5 was identified as a key downstream factor of S100A9 causing the EMT process (Figure 5I). Furthermore, ectopic expression of PGAM5 rescued the effects of S100A9 knockout and promoted tumor metastasis in vivo (Figure 5J,K). Collectively, these results demonstrate that S100A9 promotes HCC growth and metastasis via PGAM5.

### 2.7 S100A9 Is a Novel Therapeutic Target in HCC Patients

**Figure 6:**
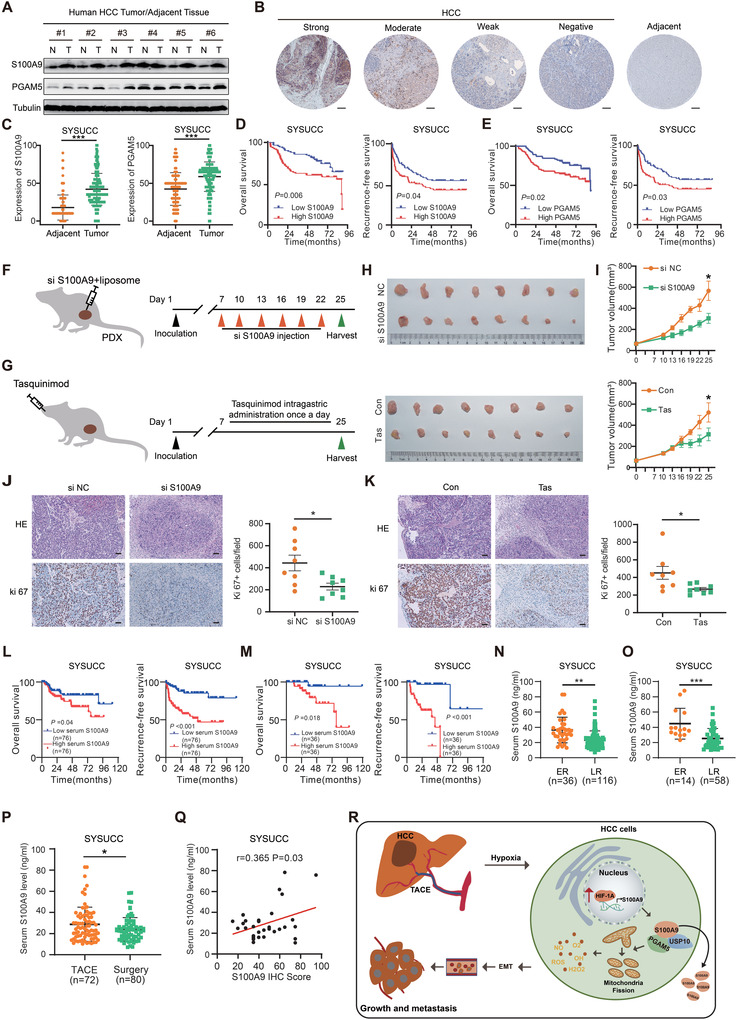
Clinical significance of S100A9 inhibition in HCC. A) Western blot analysis showing the expression of S100A9 and PGAM5 in HCC and adjacent normal tissues. B) IHC staining showing strong, moderate, weak positive, and negative expression of S100A9 in HCC and adjacent normal tissues. Scale bars = 100 µm. C) IHC score of S100A9 and PGAM5 in HCC and adjacent normal tissue in the SYSUCC cohort (*n* = 172). Data are presented as mean ± SEM, ***p < 0.001 by two‐tailed unpaired Student *t*‐test. D) Kaplan–Meier curve analysis of OS and RFS in HCC patients by the expression of S100A9 (*n* = 172) (all patients eventually underwent surgical resection of liver tumors, including a proportion of patients who had TACE treatment prior to resection) (grouping by median S100A9 expression based on IHC scores). E) Survival analysis of OS and RFS in HCC patients by the expression of PGAM5. F,G). Schematic diagram of targeting S100A9 by siRNA (F) or Tas treatment (G) in vivo (nude mice, *n* = 8 each group). H) Inhibition of S100A9 by RNAi (top) and Tas treatment (bottom) in HCC‐PDXs. I) Tumor growth curve of HCC‐PDXs treated with in vivo‐optimized siRNAs (top) and Tas (bottom). J,K) HE and Ki67 staining in HCC‐PDX tumors treated with in vivo‐optimized siRNAs (J) or Tas (K) and quantification of Ki67‐positive cells in tumors. Scale bars = 50 µm. Data in (I)–(K) are presented as mean ± SEM, *n* = 8, *p < 0.05, by two‐tailed unpaired Student *t*‐test. L) Kaplan–Meier analysis of OS and RFS in HCC patients by the level of serum S100A9 (*n* = 152) (grouping by median serum S100A9). M) Kaplan–Meier analysis of OS and RFS in HCC patients who received preoperative TACE by the level of serum S100A9 (*n* = 72). N) Serum S100A9 levels in HCC patients with ER (early recurrence) and LR (late recurrence) (*n* = 152). O) Serum S100A9 levels in HCC patients with ER and LR who received preoperative TACE (*n* = 72). P) Serum S100A9 levels in patients with treatment of preoperative TACE and surgery. Data in (N)–(P) are presented as mean ± SEM, *p < 0.05, **p < 0.01, ***p < 0.001 by two‐tailed unpaired Student *t*‐test. Q) Positive correlation analysis between IHC scores of S100A9 and preoperative serum S100A9 levels in the same patients (*n* = 36). *r* and *p* value were measured using Pearson's correlation test. R) Schematic diagram of the underlying mechanisms of TACE and S100A9. S100A9 is upregulated by TACE‐induced HIF1A, leading to increased levels of PGAM5 and subsequent ROS levels, which promote HCC tumor progression and migration.

To validate the expression pattern of S100A9 and PGAM5 in HCC, the expression of S100A9 and PGAM5 in HCC tissues and adjacent to normal liver tissues were assessed. S100A9 and PGAM5 expression was significantly upregulated in tumor tissues compared with normal liver tissues (**Figure** **6**A–C). Survival analyses showed that that HCC patients with higher S100A9 or PGAM5 levels had worse overall survival (OS) and recurrence‐free survival (RFS) (Figure 6D,E). Both univariate and multivariate analyses indicated that S100A9 is an independent risk factor for patients with HCC (Tables S1 and S2, Supporting Information). Higher S100A9 levels are associated with higher AFP levels, pathological stages, and larger tumor sizes (Figure S6A–C, Supporting Information). Similarly, higher S100A9 expression was correlated with higher T stage, tumor stage, and tumor grade and worse OS in the TCGA‐LIHC database (Figure S6D–G, Supporting Information).

To verify whether S100A9 serves as a potential therapeutic target for HCC, we constructed HCC patient‐derived xenograft (PDX) models in nude mice and utilized siRNA or Tas to target S100A9 (Figure 6F,G). Notably, inhibition of S100A9 by in vivo‐optimized RNAi or Tas administration significantly attenuated HCC‐PDX tumor growth (Figure 6H–K and Figure S6I–L, Supporting Information). Similar to previous reports,^[^ ^25^, ^26^ ^]^ a selective *β*2 blocker, but not a selective *β*1 blocker, inhibited the lung metastasis of HCC (Figure S6H, Supporting Information).

Previous reports have indicated that S100A9 acts as a secreted protein that promotes cancer development. This raises the possibility that serum S100A9 levels could predict the prognosis of HCC patients. As expected, HCC patients with higher serum S100A9 levels had worse OS and RFS (Figure 6L,M). Serum S100A9 levels were found to be higher in patients with ER than in those with LR, regardless of whether the patient had preoperative TACE (Figure 6N,O). Patients who underwent preoperative TACE tended to have higher serum S100A9 levels than those who underwent surgery alone (Figure 6P). In addition, a positive correlation between IHC scores and preoperative serum S100A9 levels was found in the same patients (Figure 6Q), indicating that serum S100A9 levels could reflect S100A9 expression in HCC. Collectively, these results indicate that the S100A9 level in HCC tissues as well as in serum may be a good predictor for HCC patients’ survival.

## 3 Discussion

TACE is a common treatment modality for patients with advanced HCC.^[^ ^3^, ^4^ ^]^ However, one of the side effects is the induction of hypoxic conditions, which facilitate HIF‐driven responses in tumor cells and activate the expression of specific genes involved in angiogenesis, cell proliferation, migration, and immunosuppression.^[^ ^27^, ^28^, ^29^ ^]^ In the present study, we observed that preoperative TACE increased the level of S100A9 via hypoxia‐derived HIF1A in HCC tissues instead of immune cells. S100A9 modulated PGAM5 levels and led to mitochondrial fission and ROS production, causing the progression of HCC post‐TACE.

In addition to the impact on cancer development, the induction of S100A9 by hypoxia may play an important role in ischemic events. Upregulation of S100A8/A9 mRNA in mice brain after induction of focal cerebral ischemia suggested that S100A8/A9 contributes to the progression of ischemic injury.^[^ ^30^ ^]^ S100A9 was also found to cause cardiomyocyte death in early‐stage myocardial ischemia–reperfusion injury.^[^ ^31^ ^]^ In this study, ischemia of HCC induced by TACE led to an increase in S100A9 levels in both HCC tissues and serum, which induced post‐TACE HCC progression. Higher S100A9 levels were associated with poor survival outcomes and a higher likelihood of early recurrence, making it a promising predictor of survival in HCC patients.

Although S100A9 has previously been reported to promote ROS production,^[^ ^15^ ^]^ its intrinsic mechanisms remain underexplored. In the present study, we explored in more detail how S100A9 is involved in ROS‐related signaling pathways in HCC cells. We found S100A9 may act as a scaffold protein. PGAM5 is one of the downstream target genes of S100A9, and S100A9 maintains the stability of PGAM5, which promotes the production of ROS.

Previous studies have reported that immune cells express and secrete S100A9 and that exogenous S100A9 might promote the proliferation and invasion of HCC cells.^[^ ^32^, ^33^, ^34^ ^]^ Here, we verified that S100A9 was predominantly expressed in EPCAM‐positive cells in HCC tissues, thus playing a role in promoting the recurrence of HCC. It is possible that S100A9 secreted by immune cells play the same role, but this needs to be verified by further experiments. The focus of the present study was to reveal that the key reason for early recurrence after TACE treatment was the elevated expression of the pro‐oncogene S100A9 induced by TACE.

PGAM5, a serine/threonine phosphatase that has no apparent phosphoglycerate mutase activity but displays phosphoserine/threonine phosphatase activity, has recently been shown to play important roles in a variety of cellular processes, including the regulation of mitochondrial function and the mediation of immune signaling.^[^ ^35^, ^36^, ^37^ ^]^ We illustrated the tumor‐promoting function of PGAM5 in HCC and identified PGAM5 as the functional downstream target of S100A9. Rescue of PGAM5 in S100A9‐knockout cells mostly restored tumor growth and migration in vitro and in vivo, suggesting a direct role of PGAM5 in promoting tumor progression that is responsible for S100A9‐mediated mitochondrial fission, ROS production, and EMT programs.

Tas is a drug with selective neutralizing activity against secreted S100A8/9.^[^ ^38^, ^39^ ^]^ It has recently emerged as a therapeutic agent for cancer and has been shown to prolong the progression‐free survival prostate cancer patients.^[^ ^40^ ^]^ We are the first to discover that Tas inhibited the expression of endogenous S100A9 in tumor cells. Therapeutic application in an HCC‐PDX mouse model indicated that the pharmacological mechanism of Tas may have important implications for developing a novel treatment strategy for HCC. A recent study found that the selective *β*2‐blocker ICI‐118551 inhibited norepinephrine‐mediated secretion of S100A8/A9 and that inhibition of S100A9 by Tas or ICI‐118551 abrogated stress‐induced reactivation of dormant tumor cells.^[^ ^25^ ^]^ Consistently,^[^ ^26^ ^]^ we noticed that selective *β*2‐blockers, but not selective *β*1‐blockers, could also inhibit the lung metastasis of HCC in vitro, raising the possibility that combining *β*2‐blockers or nonselective *β* blockers with TACE might benefit HCC patients. However, further studies are required to confirm these possibilities.

In summary, our study illustrated that preoperative TACE may increase the expression of S100A9 by creating a hypoxic tumor microenvironment, leading to the formation of metastases and resulting in a high rate of tumor recurrence in HCC patients. S100A9, which is involved in multiple processes of growth and metastasis in HCC, particularly influences mitochondrial function. Elevated serum S100A9 levels post‐TACE may be associated with poor clinical outcomes. The strong prometastatic effect of S100A9 implies that patients with high serum S100A9 levels post‐TACE may represent a unique group of patients who could benefit from S100A9 inhibitor therapy. Here, we provide insights into post‐TACE HCC progression and a novel therapeutic target for HCC patients.

## 4 Experimental Section

The study was performed according to the international, national, and institutional rules considering animal experiments, clinical studies, and biodiversity rights.

All patients had signed an informed consent form and this part of the data was submitted to the ethics committee to obtain an ethical approval number. The Institutional Review Board of Sun Yat‐Sen University Cancer Center approved this study. Approval number: GZR2019‐231.

The animal study protocol was approved by Experimental Animal Ethics Committee, Sun Yat‐Sen University Cancer Center, Guangzhou, China. Approval number: L102012020090L.

### Mice Liver Tumor of Ischemia Model

For orthotopic xenograft mouse models, 4‐week‐old BALB/c male nude mice were randomly grouped (*n* = 2 per group). After anaesthetizing and exposing the liver, Hepa1‐6 cells (2 × 10^6^) in 50 *µ*L PBS solution were orthotopically injected into left lobe of the liver using a 50 *µ*L Hamilton microliter syringe, and the incision was closed using surgery suture threads with needle. 14 days later, at the experimental endpoint, an atraumatic clip was used to interrupt the arterial and portal venous blood supply to the left liver lobes for 60 min. After 60‐min ischemia, the clips were removed and the tumor tissues were harvested.

### Mice Liver Tumor of Chemo‐Embolization Model

For orthotopic xenograft mouse models, 4‐week‐old BALB/c male nude mice were randomly grouped (*n* = 3 per group). After anaesthetizing and exposing the liver, Hepa1‐6 cells (2 × 10^6^) in 50 *µ*L PBS solution were orthotopically injected into left lobe of the liver using a 50 *µ*L Hamilton microliter syringe, and the incision was closed using surgery suture threads with needle. 14 days later, at the experimental endpoint, epirubicin (3 mg kg^−1^) or cisplatin (5 mg kg^−1^) or both were injected into the hepatic artery with a special insulin needle, followed by clamping of the left lobe artery of the tumor for 1 h with a atraumatic vascular clip, after 1 h the clips were removed and the tumor tissues were harvested.

### Patient‐Derived Xenograft Mouse Model

For PDX, surgically resectable tumor specimens were obtained from patients with histologically confirmed HCC. All surgically resectable tumors were collected in accordance with the institutional review board‐approved protocols of the SYSUCC. PDX tumors were generated by transplanting small tumor fragments isolated directly from surgical specimens subcutaneously into nude mice. Each tumor was allowed to grow until 1 cm^3^, after which it was harvested. 10% of this tumor was re‐implanted in a nude mouse, and the tumor was thus propagated for three to four generations until it was used for this experiment. Five to six nude mice were randomly grouped and implanted HCC‐PDX. siCtrl, siS100A9 (20 µg siRNA per mouse equivalent) encapsulated with liposomes were injected via the intra‐tumor injection every 3 days for 20 days, while PBS/DMSO or Tas (5 mg kg^−1^) were intragastric administration into the mice until the tumor became palpable. Tumors were measured with a caliper every 3 days to analyze tumor growth. Tumor volume was calculated by the formula *V* = *ab* ^2^/2, where *a* and *b* were the tumor's length and width, respectively. At the experimental endpoint, tumor tissues were harvested and fixed with 4% PFA for the paraffin‐embedded section.

### In Vivo Lung Metastasis Model

To establish the lung metastasis model, 4‐week‐old male nude mice (*n* = 8 per group) were injected with stable PLC/PRF/5 or MHCC‐97H cells (2 × 10^6^ cells suspended in 200 µL PBS) through the tail veins. 50 days after injection, the mice were sacrificed, and the lungs were harvested for hematoxylin and eosin (H&E) staining, which was used to count the number of metastatic nodes in lung sections. To assess the effects of Tas treatment, after injection with PLC/PRF/5 cells (2 × 10^6^) via the tail veins of the nude mice, the mice were treated with Tasquinimodin 10% DMSO with 40% PEG300, 5% Tween‐80, and 45% saline at 10 mg kg^−1^ orally or with vehicle (DMSO with 40% PEG300, 5% Tween‐80, and 45% saline) daily from the 2nd to 7th week after injection. On day 50, the mice were sacrificed, and the lungs were collected. H&E staining was performed on paraffin‐embedded lungs to identify the metastatic nodes in the lungs. All BALB/c nude mice were purchased from the Medical Experimental Animal Center of Guangdong Province (China) and fed at the Animal Experimental Center of Sun Yat‐Sen University. All animal research procedures were performed according to the Animal Care and Use Ethics Committee of Sun Yat‐Sen University Cancer Center.

### In Vivo CD11b Neutralization

To establish the lung metastasis model in Immunogenic mice, 4‐week‐old male C57 mice (*n* = 5 per group) were injected with hepa 1–6 WT or hepa 1–6 S100A9 cells (2 × 10^6^ cells suspended in 200 µL PBS through the tail veins. CD11b‐neutralizing antibodies (Bioxcell BE0007) were diluted to 1 *µ*g *µ*L^−1^ concentration with InVivoPure pH 7.0 Dilution Buffer (Bioxcell IP0070) and administered i.p. at 100 µg per mouse and repeated once every 2 days.

### CRISPR Knockout Pooled Library

Human CRISPR Knockout Pooled Library was obtained from addgene (#1000000049). The library was amplified and used according to instructions as previously described.^[^ ^41^ ^]^ The MAGeCK analysis of the library sequencing data was performed by Novogene (Beijing, China).

### Human Tissue Specimens and HCC Tissue Microarray

Human HCC tissues and matched adjacent non‐tumor liver tissues were obtained from patients who received curative surgery at the Sun Yat‐sen Cancer Center (SYSUCC; Guangzhou, China). The related clinicopathological features of the enrolled patients are presented in Table S2, Supporting Information. All samples were obtained with the informed consent of the patients. This study complied with the standards of the 1975 Declaration of Helsinki and the experiments were approved by the Ethics Committee of Sun Yat‐Sen University Cancer Center. The study complied with the standards of the Declaration of Helsinki and the experiments were approved by the Ethics Committee of SYSUCC (Approval No: GZR2019‐231). Informed and consent was obtained from all patients.

### Co‐Immunoprecipitation Assay

The cells were lysed with Pierce IP lysis buffer (Thermo Fisher, USA) supplemented with protease inhibitors. 50 *µ*L of the mixture was removed and used as input, and the remaining mixture was incubated with primary anti‐S100A9 or with anti‐IgG as a negative control overnight, as described in Table S4, Supporting Information, at 4 °C. Thereafter, an appropriate volume of Protein A/G PLUS‐Agarose beads (sc‐2003, Santa Cruz Biotechnology) was added to the mixture for conjugation for another 1 h at 4 °C. After that, the beads were washed with lysis buffer, and the precipitated proteins binding on the beads were collected. Finally, the beads were resuspended in electrophoresis loading buffer and boiled for western blotting assays.

### Multiple Immunofluorescence

For MIF, tissue specimen slides were deparaffinized, rehydrated through an alcohol series followed by antigen retrieval with sodium citrate buffer.

Tumor sections were blocked with 5% normal goat serum (Vector) with 0.1% Triton X‐100 and 3% H2O2 in PBS for 60 min at room temperature. Then, slides were incubated with the first primary antibody (diluted with PBS appropriately) overnight, objective tissue was covered with secondary antibody (appropriately respond to first primary antibody in species), and incubated at room temperature for 50 min in dark condition. This procedure was repeated for incubation of different fiprimary antibodies and secondary antibodies with corresponding properties, and then incubated with DAPI solution at room temperature for 10 min. Then images were detected and collected by slice scanner.

### Mitochondrial Morphology

Mitochondrial morphology was visualized by Mito‐Tracker Red staining using a confocal microscopy. Briefly, HCC cells were collected and seeded to a confocal dish, and cultured overnight. Subsequently, cells were fixed in 4% paraformaldehyde for 30 min and incubated with a Mito‐Tracker Red CMXRos (Beyotime, Shanghai, China) for 30 min. The immunofluorescence images were taken with an Olympus FV 1000 laser‐scanning confocal microscope (Olympus Corporation, Tokyo, Japan).

### Detection of ROS or Mitosox

ROS or Mitosox were detected by the fluorescent probe DCFH‐DA or Mitosox according to the manufacturer's protocols. Briefly, DCFH‐DA or Mitosox was diluted to a final concentration of 10 µm with serum free medium. Then cell suspension was incubated with DCFH‐DA or Mitosox at 37 °C for 30 min. The fluorescence in each group was assessed by flow cytometry.

### Single Cell Suspensions and Fluorescence Activated Cell Sorting

The preparation of single cell suspensions from tumor tissue was finished according to the manufacturer's protocols (Meltenyi biotec # 130‐095‐929). After obtaining the single cell suspensions, cells were incubated with Red Blood Cell Lysis Buffer (Solarbio R1010) for 20 min, centrifuged at 300 × *g* for 5 min, and then supernatant was aspirated completely. Thereafter, the cells were incubated with Zombie NIR Fixable Viability Kit for 15 min and then FITC‐Epcam and PC5.5‐CD45 antibodies for 1 h. Then cells were washed with ice‐cold PBS and then were fixed with 4% paraformaldehyde for 15 min and methyl alcohol for another 15 min. After that, cells were incubated with APC‐S100A9 for 1 h. The fluorescence in each group was assessed by flow cytometry.

### Seahorse

Cells were seeded to a 24‐well Seahorse XF Cell Culture Microplate (60 000 cells per well). After 24 h, the cell culture growth medium was changed to a pre‐warmed assay medium and the cell culture microplate was placed into a 37 °C non‐CO_2_ incubator for 45 min. The oxygen consumption rate and glycolysis (ECAR) was measured in the XF Analyzer according to the manual of Seahorse XF Cell Mito Stress Test Assay (Seahorse Bioscience of Agilent Technologies).

### Enzyme‐Linked Immunosorbent Assay

The enzyme‐linked immunosorbent assay procedure was finished according to the manufacturer's protocols. Briefly, 100 µL of standard and serum were added per well and incubated for 2 h at 37 °C. After that, the liquid of each well was removed and 100 µL of Biotin‐antibody was added to each well and incubated for 1 h at 37 °C. Then each well was washed three times and 100 µL of HRP‐avidin was added to each well and incubated for 1 h at 37 °C. Next, 90 µL of TMB substrate was added to each well and incubated for 15–30 min at 37 °C, then 50 µL of Stop Solution was added to each well and the optical density of each well was measured by Biotek Epoch 2 machine (BioTek, Winooski, USA) at 450 nm.

### Bioinformatics Analysis

Data from TCGA (https://portal.gdc.cancer.gov/) or RNA‐Seq were analyzed by R (V3.3, http://www.bioconductor.org) with the edge R package. FC of gene expression was calculated with threshold criteria of FC ≥2 and *p* value <0.05. KEGG pathway enrichment analysis was performed to investigate the processes of the candidate genes or metabolites, by applying online tools of the DAVID (https://david.ncifcrf.gov/). The pathway network was structured by Cytoscape 3.7.1. The online database of R2: Genomics Analysis and Visualization Platform (https://hgserver1.amc.nl) was applied to determine the clinical survival of the genes.

### Statistical Analysis

Statistical analysis was performed by GraphPad Prism 8.0 software (GraphPad, Inc., La Jolla, CA, USA). Data were analyzed by one‐way ANOVA, Student's *t*‐test, or Chi Square with at least three independent experiments and were presented as the mean ± SEM. The sample size (*n*) for each statistical analysis is indicated in detail in figure legends. Survival curves were constructed using the Kaplan–Meier method and analyzed by the log‐rank test. Significant prognostic factors found by univariate analysis were entered into a multivariate analysis using the Cox proportional hazards model. The correlations analysis was assessed by Pearson's correlation analysis. Differences between values were considered statistically significant when *p < 0.05, **p < 0.01, or ***p < 0.001.

Other methods have been reported previously.^[^ ^42^ ^]^

## Conflict of Interest

The authors declare no conflict of interest.

## Author Contributions

C.Z., Y.N., and W.L. contributed equally to this work. C.Z. performed most of the cellular, biochemical, and animal experiments. Y.N. partially designed the experimental plans and contributed to the animal experiments. W.L. obtained clinical samples and contributed to bioinformatics analysis. Yi.Y., Z.Q., and Ka.L. partially contributed to the biochemical and animal experiments. W.H., C.W., Z.H., and Y.S. partially contributed to the biochemical experiments. D.Z., Y.L., Ke.L., Y.Z., and Z.Y. provided the technological supports for animal experiments. B.L. and Yu.Y. supervised the project, designed experiments, analyzed results, and wrote the paper.

## Supporting information

Supporting Information

## Data Availability

The data that support the findings of this study are available from the corresponding author upon reasonable request.
