# Supplementary material for: S100A9 Derived from Chemoembolization‐Induced Hypoxia Governs Mitochondrial Function in Hepatocellular Carcinoma Progression
Source: Adv Sci (Weinh). 2022 Aug 30;9(30):2202206. doi: 10.1002/advs.202202206 (PMC9596847; doi:10.1002/advs.202202206)
Supplement: Supplementary file 1 — Supporting Information [file ADVS-9-2202206-s001.pdf]

ADVANCED SCIENCE
Open Access

Supporting Information

for *Adv. Sci.*, DOI 10.1002/advs.202202206

S100A9 Derived from Chemoembolization-Induced Hypoxia Governs Mitochondrial Function in Hepatocellular Carcinoma Progression

Chengrui Zhong, Yi Niu, Wenwu Liu, Yichuan Yuan, Kai Li, Yunxing Shi, Zhiyu Qiu, Keren Li, Zhu Lin, Zhenkun Huang, Dinglan Zuo, Zhiwen Yang, Yadi Liao, Yuanping Zhang, Chenwei Wang, Jiliang Qiu, Wei He, Yunfei Yuan and Binkui Li**

S100A9 derived from chemoembolization-induced hypoxia governs mitochondrial function in hepatocellular carcinoma progression

Chengrui Zhong[1,2]*, Yi Niu[1]*, Wenwu Liu[1,3]*, Yichuan Yuan[1,2], Kai Li[1,2], Yunxing Shi[1,2], Zhiyu Qiu[1,2], Keren Li[1,2], Zhu Lin[1,2], Zhenkun Huang[1,2], Dinglan Zuo[1], Zhiwen Yang[1,4], Yadi Liao[1,4], Yuanping Zhang[1,2], Chenwei Wang[1,2], Jiliang Qiu[1,2], Wei He[1,2], Yunfei Yuan[1,2#], Binkui Li[1,2#].

[1]State Key Laboratory of Oncology in South China and Collaborative Innovation Center for Cancer Medicine, Sun Yat-Sen University Cancer Center, Guangzhou, China
[2]Department of Liver Surgery, Sun Yat-Sen University Cancer Center, Guangzhou, China
[3]Department of Gastric Surgery, Sun Yat-Sen University Cancer Center, Guangzhou China
[4]Department of Anesthesiology, Sun Yat-sen University Cancer Center, Guangzhou China
*These authors contributed equally to this work.
[#]Corresponding authors: Binkui Li, E-mail: libk@sysucc.org.cn or Yunfei Yuan, E-mail: yuanyf@mail.sysu.edu.cn. Department of Liver Surgery, Sun Yat-sen University Cancer Center, 651 Dongfeng Road East, Guangzhou 510060, PR China.

Catalogue

1. **Supplementary figures 1-6 and tables 1-2**
2. **CRISPR Knockout Pooled Library.xls**
3. **Ischemia VS Con _Gene_differential_expression.xlsx**
4. **TACE_ER VS LR_Gene_differential_expression.xlsx**
5. **P-PLCPRF5-CTR-vs-P-PLCPRF5-sh1.genes. annot**
6. **Antibody, prime and chemicals reagents**

1. Supplementary figures 1-6 and tables 1-2

Supplementary figure 1

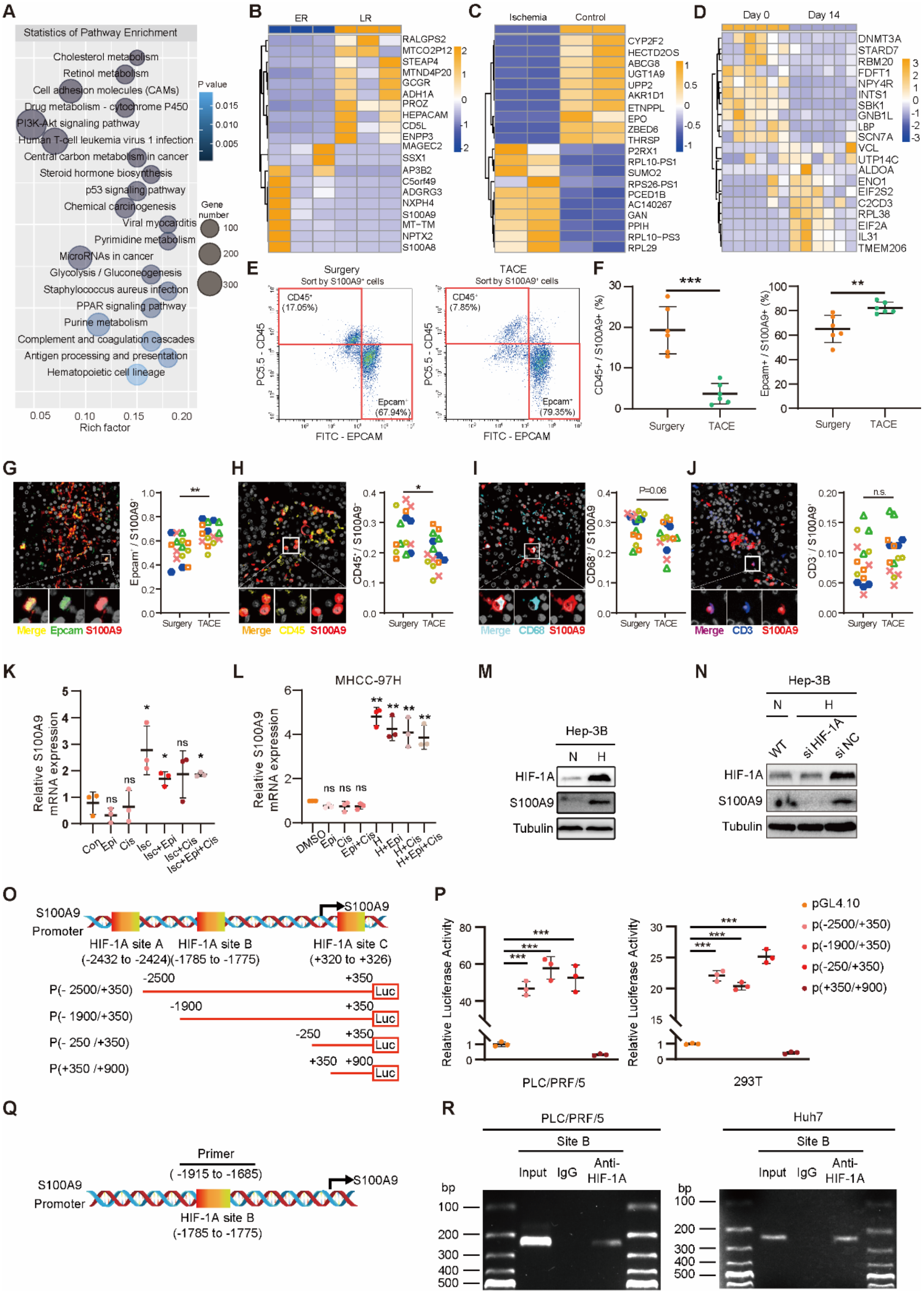

Supplementary figure 1 S100A9 is a key driver of post-TACE HCC progression.

A) KEGG enrichment analysis of DEGs in HCC tissues in patients between the early recurrence and late recurrence groups. B) Heatmap showing the DEGs between early recurrence and late recurrence (showing only the 10 most upregulated genes in each group) (tumor recurrence within two years after surgical resection). C) Heatmap showing the DEGs between the ischemia group and the control group (showing only the 10 most upregulated genes in each group). D) Heatmap showing the DEGs between day 0 and day 14 from the CRISPR/Cas9 library screen in PLC/PRF/5 cells (showing only the 10 most upregulated genes in each group). E) Flow cytometry showing the proportion of EPCAM+/S100A9+ and CD45+/S100A9+ cells among total S100A9+ cells in the HCC tissues of patients who received only surgery (top) or patients who received TACE followed by surgery (bottom). F) TACE upregulated the proportion of EPCAM+/S100A9+ cells and downregulated the proportion of CD45+/S100A9+ cells among the total S100A9+ cells in the HCC tissues of patients who received TACE followed by surgery compared to patients who received surgery only (n=6 each group). G-J) Multiple immunofluorescence staining showing DAPI (gray), S100A9 (red), EPCAM (green, Fig G), CD45 (yellow, Fig H), CD68 (bright blue, Fig I) and CD3 (blue, Fig J) expression, coexpression (double-positive cells) and quantification of each kind of cell proportion in the tumor regions in patients treated with or without TACE (three random areas for five different samples). K) RNA expression of S100A9 in orthotopic liver xenograft tumors after different treatments *in vivo*. Epi (epirubicin), Cis (cisplatinum), Isc (ischemia). L) RNA expression of S100A9 in MHCC-97H cells after different treatments *in vitro*. H (hypoxia) (1% oxygen 24 hours). M) Hypoxia stabilized HIF1A and induced S100A9 expression in Hep-3B cells. N) Silencing HIF1A suppressed the expression of S100A9 induced by hypoxia in MHCC-97H cells. O) Schematic view of the luciferase reporter constructs containing various lengths of the 5′-flanking regions of the S100A9 promoter. Detailed characterization of the S100A9 promoter by 5′-deletion and site-specific deletion analyses was performed. P) S100A9 promoter luciferase activity in PLC/PRF/5 cells or 293T cells cotransfected with the vector or the indicated luciferase reporter and pcdna3.1-HIF1A constructs for 48 h. Q) Representation of S100A9 promoter regions and the indicated primers for ChIP assay. R) ChIP assay for HIF1A occupancy at the S100A9 promoter in PLC/PRF/5 cells or Huh7 cells. Precipitated DNA was purified and subjected to semiquantitative PCR. Data in (F)-(L) and (P) is presented as mean ± SEM, * $P<0.05$, ** $P < 0.01$, *** $P < 0.001$, by two-tailed unpaired Student t-test.

Supplementary figure 2

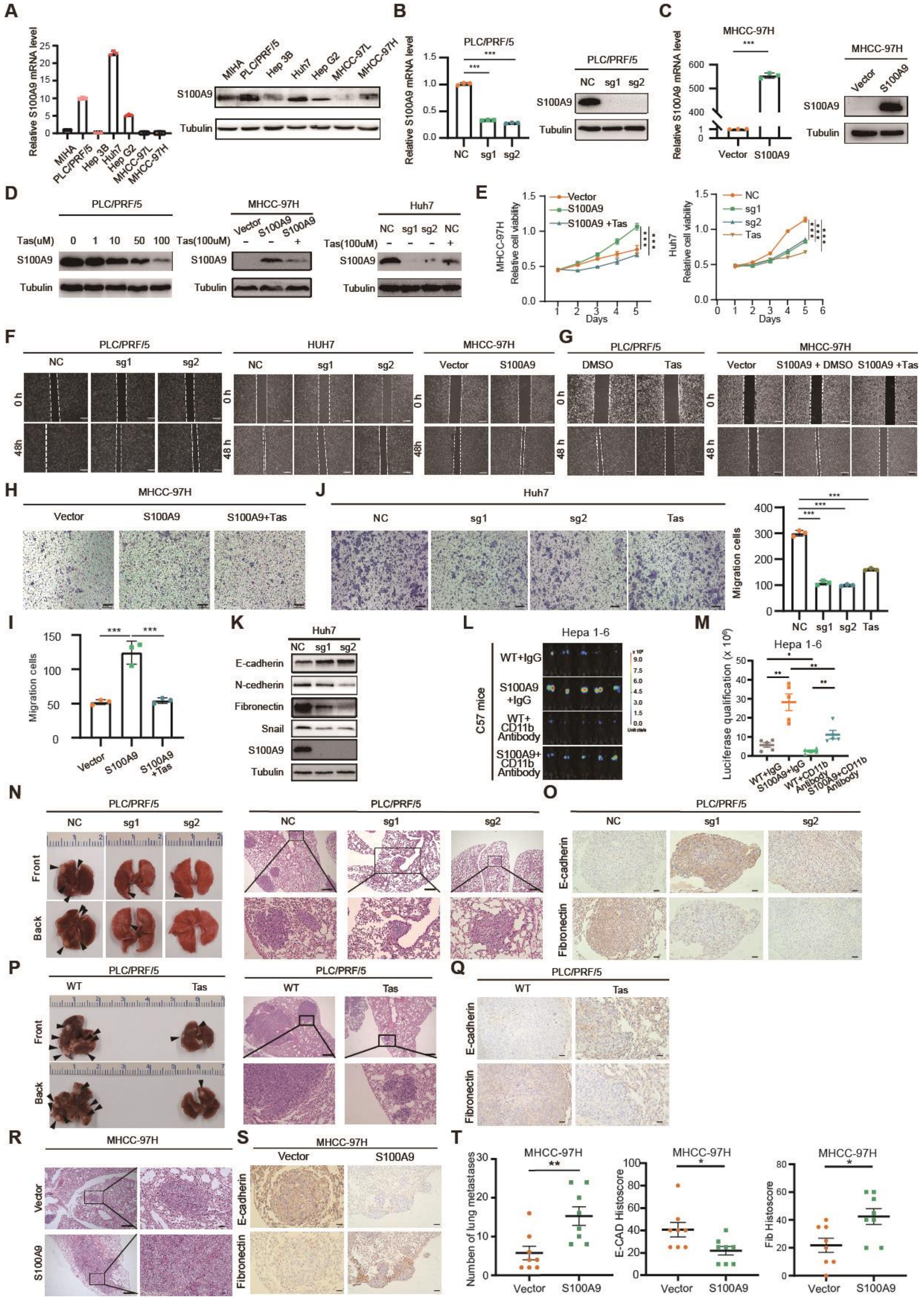

Supplementary figure 2 S100A9 promotes the growth and metastasis of HCC cells *in vitro* and *in vivo*.

A) Real-time PCR (left) and Western blotting assays (right) showing the expression of S100A9 in hepatoma cell lines(n=3). B-C) Real-time PCR and Western blot assays showing the efficiency of S100A9 knockout/overexpression in PLC/PRF/5 (B) and MHCC-97H (C) cells(n=3). D) Tas inhibited the expression of S100A9 in HCC cells. PLC/PRF/5 cells were treated with Tas at the indicated concentration for 24 h before harvest. MHCC-97H-OE and Huh7 cells were treated with 100 µM Tas for 24 h before harvesting. E) Tas inhibited the proliferation of MHCC-97H-OE (left) and Huh7 (right) cells, as indicated by CCK-8 assays(n=3). F-G) Knockout of S100A9 suppressed and overexpression of S100A9 enhanced the migration of HCC cells, as indicated by wound-healing assays. H-J) Knockout of S100A9 or 100 µM Tas inhibited the cell migration of HCC cells, as indicated by Transwell assays(n=3). K) Western blot analysis showing the expression of S100A9 and EMT markers in Huh7/S100A9-sg cells. L) Overexpression of S100A9 enhanced the metastatic ability of tumor cells, even after neutralizing CD11b-positive cells in C57 mice, which were measured by *in vivo* bioluminescent imaging (n=5 each group). M) Quantification of photon flux as the mean ± SEM. N) Knockout of S100A9 significantly suppressed the lung metastatic capacity of PLC/PRF/5. O) Representative E-cadherin and fibronectin staining in lung metastases of PLC/PRF/5-sg or control cells. P) Tas suppressed the lung metastatic capacity of PLC/PRF/5. Q) Representative E-cadherin and fibronectin staining in lung metastases of Tas-treated groups and control groups. R) Overexpression of S100A9 promoted the lung metastatic capacity of MHCC-97H cells. S) Representative E-cadherin and fibronectin staining in lung metastases of MHCC-97H-OE or control cells. T) Overexpression of S100A9 promoted the lung metastatic capacity of HCC cells and decreased E-cadherin expression and increased fibronectin expression in lung metastases (n=8 each group). Data in (A)-(E), (H)-(J), (M) and (T) are presented as mean ± SEM, * $P < 0.05$, ** $P < 0.01$, *** $P<0.001$, by two-tailed unpaired Student t-test.

Supplementary figure 3

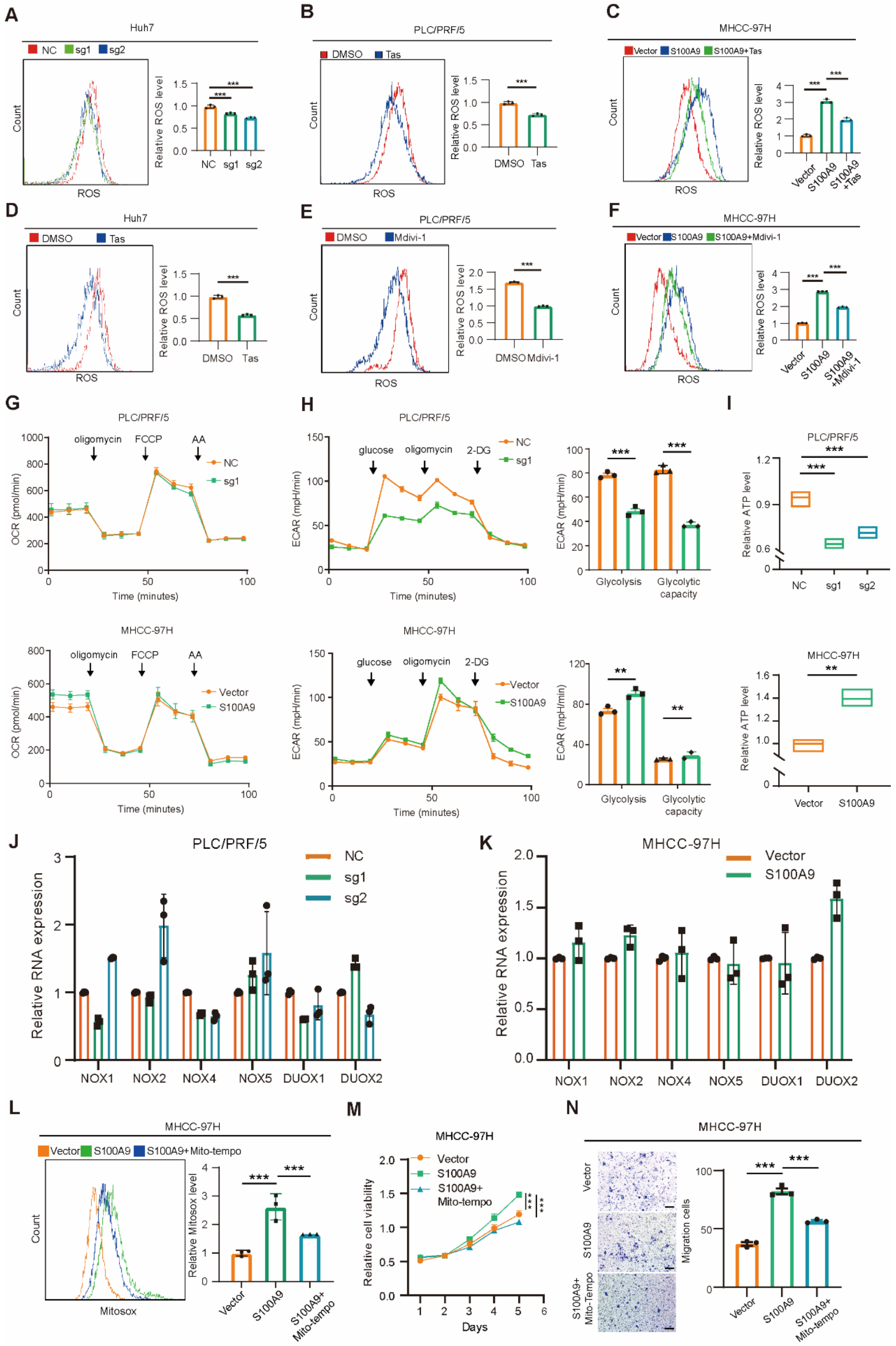

Supplementary figure 3 S100A9 modulates cellular mitochondrial function and promotes ROS production.

A.) S100A9 knockout reduced intracellular ROS production in Huh7 cells, as indicated by DCFH-DA fluorescence assay. B-D) Tas reduced intracellular ROS production in PLC/PRF/5 (B), Huh7 (C) and MHCC-97H (D) cells. E-F) Mdivil-1(10 μM) reduced intracellular ROS production in PLC/PRF/5 (E) and MHCC-97H (F) cells. G) Effects of S100A9 on oxidative phosphorylation (as determined by Seahorse XF analyzers) in PLC/PRF/5 (top) and MHCC-97H cells (bottom). The oxygen consumption rate (OCR) over time were shown. H) Effects of S100A9 on glycolytic rates (as determined by Seahorse XF analyzers) in PLC/PRF/5 (top) and MHCC-97H cells (bottom). The extracellular acidification rate (ECAR) (left) over time and the calculated glycolytic rates(right) were shown. I) S100A9 knockout reduced whereas S100A9 overexpression increased ATP production in HCC cells. J-K) Knockout of S100A9 or overexpression of S100A9 did not consistently change the RNA levels of the NOX family, as indicated by real-time PCR. L) Mito-tempo (20 μM) reduced intramitochondrial ROS production in MHCC-97H/S100A9-OE cells. M) Decreased intramitochondrial ROS with 20 μM Mito-tempo inhibited the proliferation ability of MHCC-97H/S100A9-OE cells. N) Decreased intramitochondrial ROS with 20 μM Mito-tempo inhibited the migration ability of MHCC-97H/S100A9-OE cells. Data in (A)-(N) are presented as mean ± SEM, n=3, * $P < 0.05$, ** $P < 0.01$, ***$P<0.001$, by two-tailed unpaired Student t-test.

Supplementary figure 4

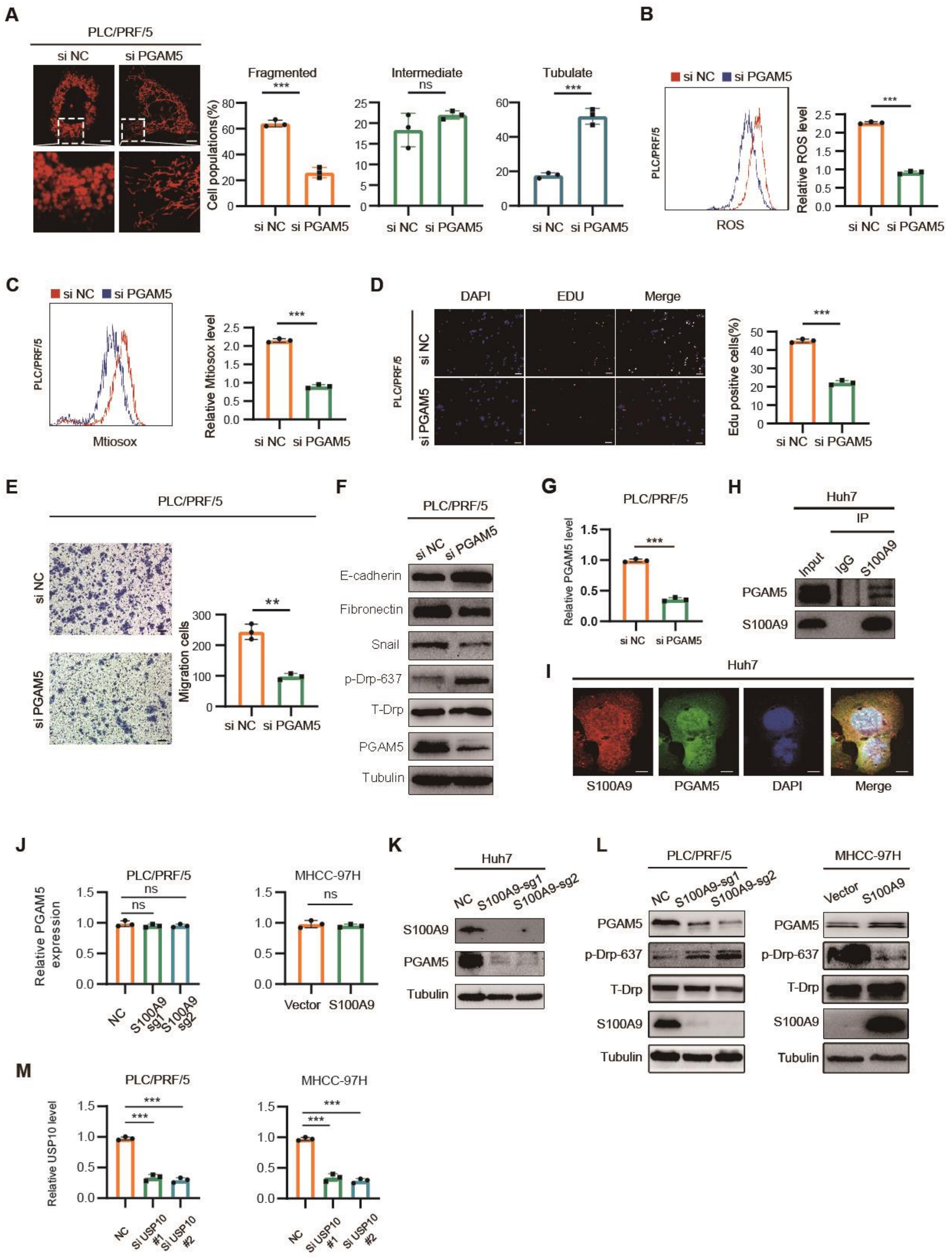

Supplementary figure 4 S100A9 recruits USP10 and PGAM5 to maintain PGAM5 stability.

A) Knockdown of PGAM5 inhibited mitochondrial fission in PLC/PRF/5(up) cells. Scale bars, 20 μm. The proportion of HCC cells (n =100 cells for each sample, repeated three times) with tubulated, intermediate, and fragmented mitochondria was quantified. B) Knockdown of PGAM5 inhibited intracellular ROS production in PLC/PRF/5 cells, as indicated by DCFH-DA fluorescence assay. C) Knockdown of PGAM5 inhibited intramitochondrial ROS production in PLC/PRF/5 cells, as indicated by the MitoSOX fluorescence assay. D) Knockdown of PGAM5 inhibited the proliferation of PLC/PRF/5 cells, as indicated by EdU assay. E) Knockdown of PGAM5 inhibited the migration of PLC/PRF/5 cells, as indicated by Transwell assays. F) Western blot for PGAM5 and PGAM5 target genes and EMT markers in HCC cells. G) Real-time PCR showing the knockdown efficiency of PGAM5 in PLC/PRF/5 cells. H) Co-IP showing the interaction of S100A9 and PGAM5 in Huh7 cells. I) Representative images of immunofluorescence colocalization of S100A9 (red) and PGAM5 (green) in Huh7 cells. J) Real-time PCR showed that S100A9 did not change the RNA level of PGAM5 in HCC cells. K) Knockout of S100A9 decreased the protein level of PGAM5 in Huh7 cells. L) Western blot showing the expression of S100A9 and PGAM5 target genes in PLC/PRF/5 and MHCC-97H cells. S100A9 knockout increased while S100A9 overexpression decreased dephosphorylation at Ser637 of DRP. M) Real-time PCR showing the knockdown efficiency of USP10 in HCC cells. Data in (A)–(E), (G), (J), (M) are presented as mean ± SEM, n = 3. n.s. none sense. ** $P < 0.01$, *** $P < 0.001$ by two-tailed unpaired Student t-test.

Supplementary figure 5

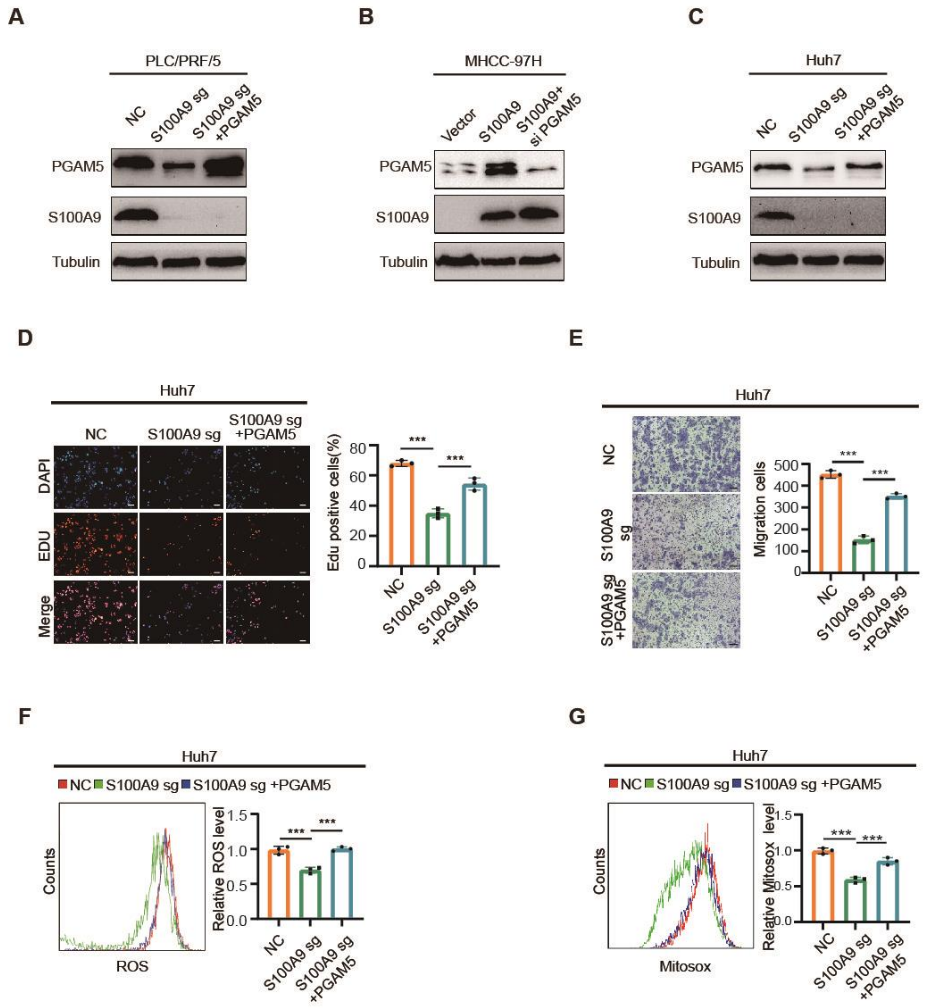

Supplementary figure 5 S100A9 affects HCC growth and metastasis through a PGAM5-dependent pathway.

A) Western blot analysis showing the PGAM5 overexpression efficiency in PLC/PRF/5 cells. B) Western blot analysis showing the PGAM5 knockdown efficiency in MHCC-97H cells. C) Western blot analysis showing the PGAM5 overexpression efficiency in Huh7 cells. D) Ectopic expression of PGAM5 enhanced the growth of S100A9-sg Huh7 cells, as indicated by EdU assay. E) Ectopic expression of PGAM5 enhanced the migration of S100A9-sg Huh7 cells, as indicated by the Transwell assay. F) Ectopic expression of PGAM5 enhanced the intracellular ROS level of Huh7-S100A9-sg cells, as indicated by DCFH-DA fluorescence assay. G) Ectopic expression of PGAM5 enhanced the intramitochondrial ROS level of Huh7-S100A9-sg cells, as indicated by the MitoSOX fluorescence assay. Data in (E)–(G) are presented as mean ± SEM, n = 3. *** $P < 0.001$ by two-tailed unpaired Student t-test.

Supplementary figure 6

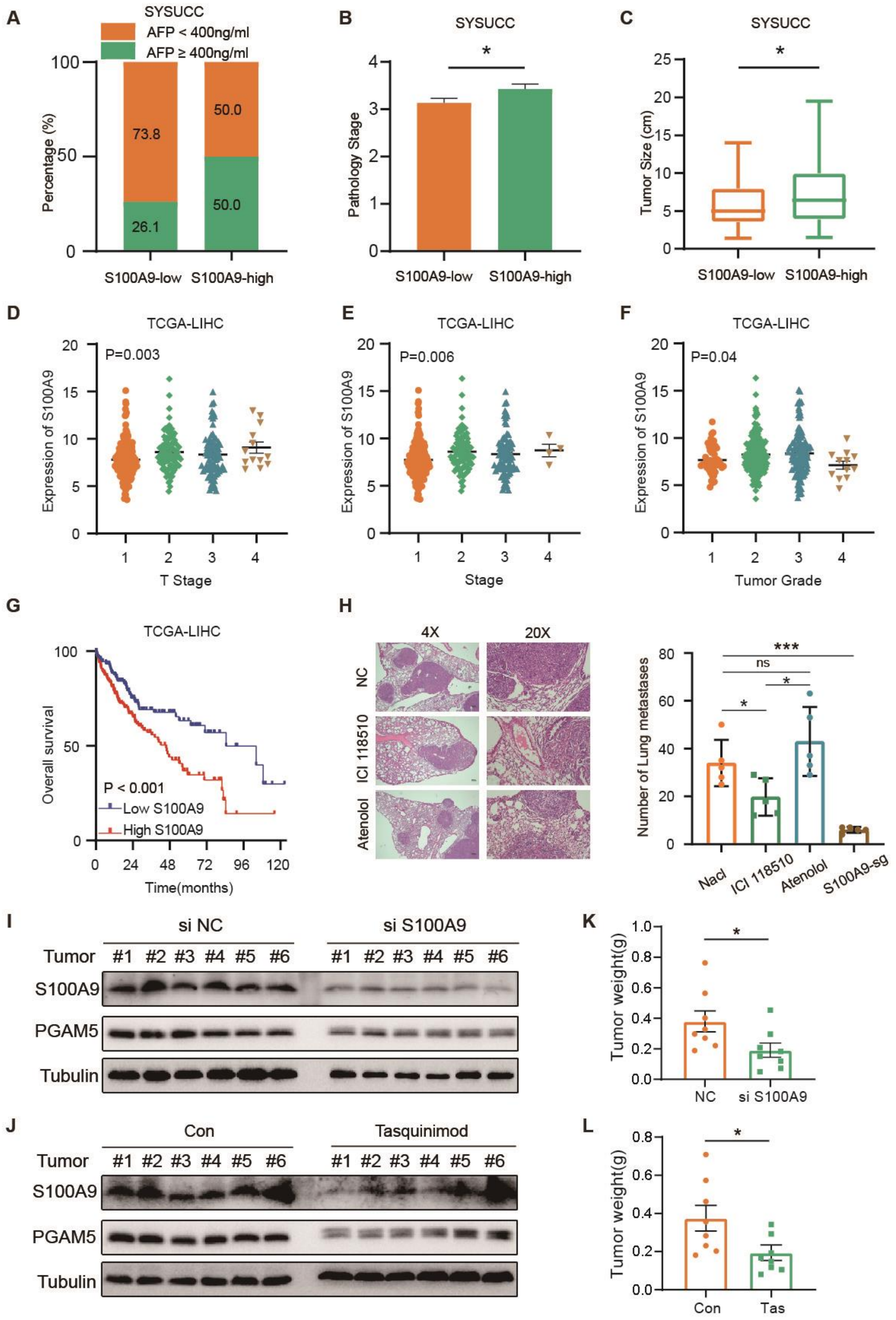

Supplementary figure 6 Clinical significance of S100A9 inhibition in HCC.

A) Stacked histogram for AFP levels in the S100A9-High group and S100A9-Low group in the SYSUCC cohort. B) Correlation between S100A9 expression and pathological stage in the SYSUCC cohort (n=172) (grouping by median S100A9 expression based on IHC score). C) Correlation between S100A9 expression and tumor size in the SYSUCC cohort (n=172) (grouping by median S100A9 expression based on IHC score). D-F) Correlation between S100A9 expression and T stage, tumor stage and tumor grade in the TCGA-LIHC cohort. Data in (D)–(F) are presented as mean ± SEM. n=352. Significant differences were determined by one-way ANOVA. G) Kaplan–Meier curve analysis of OS in HCC patients by the expression of S100A9 in the TCGA-LIHC cohort (n=352) (grouping by median S100A9 expression). H) Representative HE staining of lung metastases (left) and quantification of the tumor number of lung metastases (right) of PLC/PRF/5 cells treated with different drugs as indicated. Data in (H) are presented as mean ± SEM. n=5. n.s. none sense, * P < 0.05, *** P<0.001 by one-way ANOVA. I) Western blot analysis showing the inhibition efficiency of S100A9 by RNAi in HCC-PDXs. J) Western blot analysis showing the inhibition efficiency of Tas in HCC-PDXs. K) Tumor weight of HCC-PDXs treated with *in vivo*-optimized siRNA. L) Tumor weight of HCC-PDXs treated with Tas. Data in (K) and (L) are presented as mean ± SEM, n = 6. * P < 0.05 by two-tailed unpaired Student t-test.

Supplementary Table 1 Correlation between S100A9 and clinicopathological parameters

Characteristics	S100A9 low (n = 88)	S100A9 High (n = 84)	P value
Age			
≤50	43(48.8%)	41(48.8%)	0.994
>50	45(51.2%)	43(51.2%)	
Sex			0.438
Male	77(87.5%)	70(83.3%)	
Female	11(12.5%)	14(16.7%)	
HBV			0.928
Yes	81(92.0%)	77(91.7%)	
No	7(8.0%)	7(8.3%)	
AFP			0.001
≥400 ng/ml	23(26.1%)	42(50%)	
<400 ng/ml	65(73.9%)	42(50%)	
Tumour size			0.042
<10cm	75(85.2%)	61(72.6%)	
≥10cm	13(14.8%)	23(27.4%)	
Complete capsule			0.888
Yes	15(17.0%)	15(17.9%)	
No	73(83.0%)	69(82.1%)	
Microvascular invasion			0.864
Yes	23(26.1%)	21(25%)	
No	65(73.9%)	63(75%)	
Margin			0.004

Clear	85(96.6%)	70(83.3%)	
unclear	3(3.4%)	14(16.7%)	
Cirrhosis (yes/no)			0.172
Yes	66(75.0%)	55(65.5%)	
No	22(25.0%)	29(34.5%)	
CA19_9			0.294
≥35 U/mL	20(22.7%)	25(29.8%)	
<35 U/mL	68(77.3%)	59(70.2%)	
CEA			0.101
≥5 ng/mL	16(18.2%)	8(9.5%)	
<5 ng/mL	72(81.8%)	76(90.5%)	
ALB			0.341
>35 g/L	83(94.3%)	76(90.5%)	
≤35 g/L	5(5.7%)	8(9.5%)	
ALT			0.703
>50U/L	29(33.0%)	30(35.7%)	
≤50 U/L	59(67.0%)	54(64.3%)	
AST			0.903
>40U/L	39(44.3%)	38(45.2%)	
≤40U/L	49(55.7%)	46(54.8%)	
TBIL			0.084
>17.1μmol/L	28(31.8%)	17(20.2%)	
≤17.1μmol/L	60(68.2%)	67(79.8%)	

HBV, Hepatitis B virus; AFP, α-fetoprotein; CA19-9, Carbohydrate antigen 19-9; CEA, Carcinoma Embryonic Antigen; ALB, Albumin; ALT, Alanine aminotransferase; AST, Aspartate aminotransferase; TBIL, Total bilirubin

Supplementary Table 2 Univariate and Multivariate Cox analysis of factors associate with overall survival of HCC patients in SYSUCC cohort

Characteristic	Univariate		Multivariate	
	HR (95% CI)	P	HR (95% CI)	P
Age>50 (yes/no)	0.844(0.498-1.431)	0.530		
Sex (male/female）	1.205(0.545-2.662)	0.645		
Tumor size(>10cm) (yes/no)	1.888(0.995-3.582)	0.052		
Margin unclarity (yes/no)	1.758(0.884-3.494)	0.108		
Complete capsule (yes/no)	1.366(0.663-2.818)	0.398		
Microvascular invasion (yes/no)	1.818(1.049-3.151)	0.033	1.845(1.060-3.211)	0.03
HBV infection (yes/no)	0.619(0.265-1.449)	0.269		
IHC scores of S100A9 (high/low)	1.956(1.136-3.370)	0.016	2.057(1.192-3.548)	0.01
IHC scores of PGAM5 (high/low)	1,891(1.081-3.308)	0.025		
AFP >400 ng/ml (yes/no）	1.496(0.882-2.536)	0.135		
CA19_9 > 35 U/mL (yes/no)	1.349(0.769-2.367)	0.296		
CEA > 5 ng/mL (yes/no)	0.671(0.287-1.568)	0.357		
Cirrhosis (yes/no)	0.830(0.471-1.464)	0.52		
ALB > 35 g/L (yes/no)	0.562(0.238-1.164)	0.113		
ALT > 50U/L (yes/no)	2.340(1.384-3.956)	0.002	2.445(1.443-4.144)	0.001
AST > 40U/L (yes/no)	2.339(1.365-4.008)	0.002		
TBIL > 17.1μmol/L (yes/no)	1.300(0.724-2.334)	0.379		

HR, Hazard ratio; CI, Confidence interval; HBV, Hepatitis B virus; AFP, α-fetoprotein; CA19-9, Carbohydrate antigen 19-9; CEA, Carcinoma Embryonic Antigen; ALB, Albumin; ALT, Alanine aminotransferase; AST, Aspartate aminotransferase; TBIL, Total bilirubin

Supplementary Table 1 Correlation between S100A9 and clinicopathological parameters

Characteristics	S100A9 low (n = 88)	S100A9 High (n = 84)	P value
Age			
≤50	43(48.8%)	41(48.8%)	0.994
>50	45(51.2%)	43(51.2%)	

Sex			0.438
Male	77(87.5%)	70(83.3%)	
Female	11(12.5%)	14(16.7%)	
HBV			0.928
Yes	81(92.0%)	77(91.7%)	
No	7(8.0%)	7(8.3%)	
AFP			0.001
≥400 ng/ml	23(26.1%)	42(50%)	
<400 ng/ml	65(73.9%)	42(50%)	
Tumour size			0.042
<10cm	75(85.2%)	61(72.6%)	
≥10cm	13(14.8%)	23(27.4%)	
Complete capsule			0.888
Yes	15(17.0%)	15(17.9%)	
No	73(83.0%)	69(82.1%)	
Microvascular invasion			0.864
Yes	23(26.1%)	21(25%)	
No	65(73.9%)	63(75%)	
Margin			0.004
Clear	85(96.6%)	70(83.3%)	
unclear	3(3.4%)	14(16.7%)	
Cirrhosis (yes/no)			0.172
Yes	66(75.0%)	55(65.5%)	
No	22(25.0%)	29(34.5%)	
CA19_9			0.294
≥35 U/mL	20(22.7%)	25(29.8%)	
<35 U/mL	68(77.3%)	59(70.2%)	
CEA			0.101
≥5 ng/mL	16(18.2%)	8(9.5%)	
<5 ng/mL	72(81.8%)	76(90.5%)	
ALB			0.341
>35 g/L	83(94.3%)	76(90.5%)	
≤35 g/L	5(5.7%)	8(9.5%)	
ALT			0.703
>50U/L	29(33.0%)	30(35.7%)	
≤50 U/L	59(67.0%)	54(64.3%)	
AST			0.903
>40U/L	39(44.3%)	38(45.2%)	
≤40U/L	49(55.7%)	46(54.8%)	

TBIL			0.084
>17.1μmol/L	28(31.8%)	17(20.2%)	
≤17.1μmol/L	60(68.2%)	67(79.8%)	

HBV, Hepatitis B virus; AFP, α-fetoprotein; CA19-9, Carbohydrate antigen 19-9; CEA, Carcinoma Embryonic Antigen; ALB, Albumin; ALT, Alanine aminotransferase; AST, Aspartate aminotransferase; TBIL, Total bilirubin

Supplementary Table 2 Univariate and Multivariate Cox analysis of factors associate with overall survival of HCC patients in SYSUCC cohort

Characteristic	Univariate		Multivariate	
	HR (95% CI)	P	HR (95% CI)	P
Age>50 (yes/no)	0.844(0.498-1.431)	0.530		
Sex (male/female)	1.205(0.545-2.662)	0.645		
Tumor size(>10cm) (yes/no)	1.888(0.995-3.582)	0.052		
Margin unclarity (yes/no)	1.758(0.884-3.494)	0.108		
Complete capsule (yes/no)	1.366(0.663-2.818)	0.398		
Microvascular invasion (yes/no)	1.818(1.049-3.151)	0.033	1.845(1.060-3.211)	0.03
HBV infection (yes/no)	0.619(0.265-1.449)	0.269		
IHC scores of S100A9 (high/low)	1.956(1.136-3.370)	0.016	2.057(1.192-3.548)	0.01
IHC scores of PGAM5 (high/low)	1,891(1.081-3.308)	0.025		
AFP >400 ng/ml (yes/no)	1.496(0.882-2.536)	0.135		
CA19_9 > 35 U/mL (yes/no)	1.349(0.769-2.367)	0.296		
CEA > 5 ng/mL (yes/no)	0.671(0.287-1.568)	0.357		
Cirrhosis (yes/no)	0.830(0.471-1.464)	0.52		
ALB > 35 g/L (yes/no)	0.562(0.238-1.164)	0.113		
ALT > 50U/L (yes/no)	2.340(1.384-3.956)	0.002	2.445(1.443-4.144)	0.001
AST > 40U/L (yes/no)	2.339(1.365-4.008)	0.002		
TBIL > 17.1μmol/L (yes/no)	1.300(0.724-2.334)	0.379		

HR, Hazard ratio; CI, Confidence interval; HBV, Hepatitis B virus; AFP, α-fetoprotein; CA19-9, Carbohydrate antigen 19-9; CEA, Carcinoma Embryonic Antigen; ALB, Albumin; ALT, Alanine aminotransferase; AST, Aspartate aminotransferase; TBIL, Total bilirubin

2. CRISPR Knockout Pooled Library.xls

This document has been uploaded as a separate file.

3. Ischemia VS Con _Gene_differential_expression.xlsx

This document has been uploaded as a separate file.

4. TACE_ER VS LR_Gene_differential_expression.xlsx

This document has been uploaded as a separate file.

5. P-PLCPRF5-CTR-vs-P-PLCPRF5-sh1.genes.annot

6. Antibody, prime and chemicals reagents

Antibodies	Company sources	Identifier
Rabbit anti- S100A9 (CO-IP)	Cell signaling technology	Cat # 72590
Rabbit anti- S100A9 (IB)	Proteintech	Cat # 26992-1-AP
Rabbit anti- S100A9 (IHC, IF)	Abcam	Cat # ab63818
Rabbit anti- HIF1A	Proteintech	Cat # 20960-1-AP
Rabbit anti- DRP1	Cell signaling technology	Cat # 8570
Rabbit anti- DRP1 (Ser637)	Cell signaling technology	Cat # 4867
Rabbit anti- PGAM5(IB)	Cell signaling technology	Cat # 24584
Mouse anti- PGAM5(IHC, IF)	Santa Cruz	Cat # sc-515880
Mouse anti- E-cadherin	BD Transduction	Cat # 610181
Rabbit anti- N-Cadherin	Cell signaling technology	Cat # 13116
Rabbit anti- Snail	Cell signaling technology	Cat # 3879
Rabbit anti- Fibronectin	Abcam	Cat # ab268020
Rabbit anti- Ki67	Abcam	Cat #ab15580
Rabbit anti- HA-Tag	Cell signaling technology	Cat # 3724
Rabbit anti- His-Tag	Cell signaling technology	Cat # 12698
Mouse anti- Myc-Tag	Cell signaling technology	Cat # 2276
Rabbit anti- DYKDDDDK Tag	Cell signaling technology	Cat # 14793
Rabbit anti- S100A9 (MIF)	Servicebio	GB111079
Mouse Anti -EpCAM (MIF)	Servicebio	GB14078
Mouse Anti -CD45 (MIF)	Servicebio	GB14038
Rabbit Anti -CD68 (MIF)	Servicebio	GB113150
Rabbit Anti -CD3 (MIF)	Servicebio	GB11014
Rabbit anti- USP10	Proteintech	Cat # 19374-1-AP
Mouse anti- Alpha Tubulin	Proteintech	Cat # 11224-1-AP
Mouse anti- Beta Actin	Proteintech	Cat # 60008-1-Ig
Anti-rabbit IgG, HRP-linked	Cell signaling technology	Cat #7074
Anti-mouse IgG, HRP-linked	Cell signaling technology	Cat #7076
Fitc-Epcam	Invitrogen	
PC5.5-CD45	Invitrogen	
APC-S100A9	Invitrogen	
**Drugs and chemicals reagents**		
Tasquinimod	MedChemExpress	Cat # HY-10528
ICI 118,551	MedChemExpress	Cat # HY-13951
Atenolol	MedChemExpress	Cat # HY-17498
N-acetyl-L-Cysteine (NAC)	Beyotime	Cat # S0077
MitoSOX	Thermo Fisher Scientific	Cat #M36008
Mito tracker red	Beyotime	Cat #C1035
DCFH-DA	Beyotime	Cat #S0033S
Lipofectamine RNAiMAX	Invitrogen	Ca t# 13778030

Lipofectamine 3000	Invitrogen	Cat # L3000015
Mdivi-1	Beyotime	Cat # SC8028
Mito-tempo	MedChemExpress	Cat # HY-112879
Polybrene	ThermoFisher	Cat # 107689
Zombie NIR™ Fixable Viability Kit	Biolegend	Cat # 423105
Cell Lysis Buffer	Cell signaling technology	Cat # 9803S
Trizol reagent	Invitrogen	Cat # 15596018
Pierce IP lysis buffer	Invitrogen	Cat # 87787
Protein A/G PLUS-Agarose beads	Santa Cruz	Ca t# sc-2003
Puromycin	Invitrogen	Cat # A1113802
Seahorse XFe24 FluxPak	Seahorse bioscience	Cat # 102340-100
Seahorse XF Cell Mito Stress Test kit	Seahorse bioscience	Cat # 103015-100
Seahorse XF Glycolysis Stress Test kit	Seahorse bioscience	Cat # 103020-100
SimpleChIP® Enzymatic Chromatin IP Kit (Magnetic Beads)	Cell signaling technology	Cat # 9003
Migration assay	Corning	Cat # 354578
Cell counting kit	Dojindo	Cat # CK04
S100A9 ELISA Kit	CUSABIO	Cat # CSB-E11834h
Lenti-Pac HIV Expression Packaging Kit	GeneCopoeia	Cat # LT002
RNA Quick Purification kit	ESscience	Cat # RN001
ATP Colorimetric/Fluorometric Assay Kit	BioVision	Cat # K354-100
Luc-Pair™ Duo-Luciferase HS Assay Kit	GeneCopoeia	Cat # LF005
Sg Negative control GACCGGGGCGAGGAGCTGTTCACCG	Kidan Biosciences	NA
S100A9-sgRNA #1 GACTTGCAAAATGTCGCAGC	Kidan Biosciences	NA
S100A9-sgRNA #2 GCACCCAGACACCCTGAACC	Kidan Biosciences	NA
S100A9-shRNA #2 GGCCAAATAAAGTCTCTTCCT	Genepharma	NA
S100A9-shRNA #4 GCCTGTTATGTCAAACTGTCT	Genepharma	NA
si PGAM5 GUCCCUUAAAUAUUUGUCATT	Genepharma	NA
si USP10 #1 GCUUUGGAUGGAAGUUCUATT	Genepharma	NA
si USP10 #2 GCACACCACGGAAAGCAUATT	Genepharma	NA

Primer sequences		
Construct	Direction	Sequence (5’ – 3’)
S100A9(Human)	Forward	GCACCCAGACACCCTGAACCA

	Reverse	TGTGTCCAGGTCCTCCATGATG
S100A9(Mouse)	Forward	TGGTGGAAGCACAGTTGGCAAC
	Reverse	CAGCATCATACACTCCTCAAAGC
Actin (Human)	Forward	CACCATTGGCAATGAGCGGTTC
	Reverse	AGGTCTTTGCGGATGTCCACGT
PGAM5(Human)	Forward	ATCTGTCACGCCAACGTCATCC
	Reverse	CAGCAAGTGAAAGAGGTCAGGAC
USP10(Human)	Forward	AAATGCCACCGAACCTATCGGC
	Reverse	CAGCCATTCAGACCGATCTGGA
NOX1(Human)	Forward	GGTTTTACCGCTCCCAGCAGAA
	Reverse	CTTCCATGCTGAAGCCACGCTT
NOX2(Human)	Forward	CTCTGAACTTGGAGACAGGCAAA
	Reverse	CACAGCGTGATGACAACTCCAG
NOX4(Human)	Forward	GCCAGAGTATCACTACCTCCAC
	Reverse	CTCGGAGGTAAGCCAAGAGTGT
NOX5(Human)	Forward	CCACCATTGCTCGCTATGAGTG
	Reverse	GCCTTGAAGGACTCATACAGCC
DUOX1(Human)	Forward	TCTCTGGCTGACAAGGATGGCA
	Reverse	AGGCGAGACTTTTCCTCAGGAG
DUOX2(Human)	Forward	CAATGGCTACCTGTCCTTCCGA
	Reverse	GTCCTTGGAGAGGAAGCCATTC
hS100A9-chip-site A	Forward	AGGAGGTCCAAGAACATCTGGT
hS100A9-chip-site A	Reverse	GGGGCATGAGAACATCAACG
hS100A9-chip-site B	Forward	AAGCCCTGTCCTTAGCTACCA
hS100A9-chip-site B	Reverse	CGACAATACATGGAAAGTTGGGA
hS100A9-chip-site C	Forward	AGGCCATCTCCCAGTTAAGTTGC
hS100A9-chip-site C	Reverse	TGGGACACTTAGAAGAAAATGCTG

References

1. Shalem O, Sanjana NE, Hartenian E, Shi X, Scott DA, Mikkelson T, Heckl D, et al. Genome-scale CRISPR-Cas9 knockout screening in human cells. Science 2014;343:84-87.
2. Cai MY, Tong ZT, Zheng F, Liao YJ, Wang Y, Rao HL, Chen YC, et al. EZH2 protein: a promising immunomarker for the detection of hepatocellular carcinomas in liver needle biopsies. Gut 2011;60:967-976.
3. Wang C, Liao Y, He W, Zhang H, Zuo D, Liu W, Yang Z, et al. Elafin promotes tumour metastasis and attenuates the anti-metastatic effects of erlotinib via binding to EGFR in hepatocellular carcinoma. J Exp Clin Cancer Res 2021;40:113.
